# Improved FWH Path-Planning Algorithm Based on Multi-Strategy Enhancement for AUVs Operating in Coral Reef Areas

**DOI:** 10.3390/s26165079

**Published:** 2026-08-11

**Authors:** Qingjun Zeng, Xiao Feng, Hewei Xu, Xiaoqiang Dai, Yifeng Han

**Affiliations:** School of Automation, Jiangsu University of Science and Technology, Zhenjiang 212100, China

**Keywords:** path planning, IFWH, propulsion energy cost optimization, obstacle avoidance, autonomous underwater vehicle

## Abstract

In this paper, an improved fixed-width histogram (IFWH) algorithm with multi-strategy enhancement is proposed for simulation-based AUV path planning in complex underwater environments. A multi-constraint optimization framework considering propulsion energy consumption, obstacle threats, terrain collision risks, and maneuverability constraints is established. Chaotic population initialization, adaptive global–local search transfer, quadratic interpolation, and spiral local refinement are integrated to enhance population diversity and trajectory smoothness. Numerical simulations are conducted under current-free terrains, overlapping seabed-current conditions, and environments with static and dynamic obstacles. Compared with A*, FWH, IPSO, and SVF-RRT*, IFWH reduces the average terrain slope by 43.91%, 47.24%, 44.94%, and 43.71%, respectively, and decreases estimated propulsion energy consumption by 35.29%, 37.38%, 24.66%, and 35.93% in current-free Scenario A. Under overlapping seabed-current conditions, IFWH achieves a 100% success rate over 30 independent trials, obtaining average slope angles of 15.58° and 18.73° with propulsion energy costs of 1163.57 J and 1116.99 J, respectively. In mixed environments, all trials are completed without collision, with average planning times of 1.674 0.700 s and 0.692±0.053 s under following- and rotary-current conditions, respectively.

## 1. Introduction

With recent advances in marine robotics and sensing technologies, AUV has become an important platform for coral reef monitoring, including coral bleaching assessment and biodiversity surveys [1,2]. As coral bleaching events become more frequent and severe worldwide [3,4], reliable reef monitoring is essential to access the stability of the ecosystem and changes in biodiversity. Due to their mobility and autonomy, AUVs provide a promising approach to repeat large-scale coral reef observation [5].

However, path planning in coral reef environments remains challenging. Steep seabed terrain, ocean-current disturbances, dense coral communities, and limited battery capacity increase navigation risk and energy constraints [6,7,8]. Environmental disturbances, such as currents, floating debris, and moving marine organisms, may further complicate the planning problem [9]. Therefore, from the perspective of simulation-based path-planning research, it is necessary to develop a method that jointly considers propulsion energy cost estimation, terrain adaptability, and obstacle-related constraints.

In the AUV path-planning problem, the complexity of finding an optimal path increases rapidly with the size of the search space [10]. Therefore, an effective planning algorithm should have good search capability in high-dimensional and constrained environments.

As a widely used method for solving NP-hard problems, evolutionary algorithms [11,12,13,14] can be effectively adapted to complex search spaces but are computationally more resource-intensive and prone to fall into local optima. Li et al. [15] proposed an improved particle swarm optimization algorithm that incorporates adaptive inertia weights and active particles from the fish swarm algorithm to enhance global search capability. However, the method does not account for the dynamic variability of ocean currents over time, which may compromise the practical applicability of the planned path in real marine environments. Tian et al. [16] proposed a coevolutionary genetic algorithm, which is applicable to path planning in uncertain three-dimensional submarine current fields by constructing a cooperative mechanism of dual-population and a robust minimum-maximum model. However, this method has insufficient real-time capability and does not fully consider the dynamic coupling relationship between the carrier movement and the flow field. Sarkar et al. [17] proposed a genetic algorithm driven by domain knowledge, which performs exceptionally well in static environments by introducing four specific operators and combining them with an elite retention strategy. However, the algorithm lacks dynamic adaptability. Xu et al. [18] proposed a multi-objective algorithm for dimension exploration and differential evolution, which dynamically adjusts the convergence and diversity of the algorithm under different dimensions and identifies key dimensions through dimension perturbation. However, the mutation probabilities of non-critical dimensions need to be manually adjusted and lack adaptability.

Energy constraints and ocean-current disturbances are important factors in AUV path-planning research. To address these factors, recent studies have incorporated ocean-current effects into path-planning methods [19,20,21,22]. Most existing methods use simplified current models to modify the path-generation process, helping to improve the energy efficiency and feasibility of planned paths at the simulation level. However, simplified current representations may not fully describe current variations in obstacle-rich regions, especially when complex terrain or reef structures affect the local flow field. Zhang et al. [23] proposed the SVF-RRT* algorithm, which improved computational efficiency. However, this algorithm relies on flow functions to quantify the influence of ocean currents and has poor dynamic obstacle avoidance performance under simulated scenarios. Guo et al. [24] improved the artificial jellyfish search algorithm (IJS), which reduced the time cost. This algorithm optimizes the AUV path by designing an ocean current interference function. However, the fitting effect of this algorithm is poor and the path energy consumption is high. Yin et al. [25] proposed a collaborative genetic algorithm, which performed well in a simple ocean current environment. However, the algorithm did not address the coupling relationship between dynamic obstacles and ocean currents.

Although the above algorithms have made progress in path planning under complex seabed current conditions, several critical challenges remain. Specifically, due to the presence of ocean currents and the complexity of seabed obstacles, existing methods struggle to achieve low-cost trajectory optimization while maintaining terrain adaptability and obstacle constraints. To address these challenges in simulation-based path planning scenarios, we propose an improved fixed-width histogram-based estimation of distribution algorithm (IFWH). In complex simulated environments, the IFWH demonstrates the ability to adapt the planned path to varying conditions and to consider the utilization of ocean current energy to improve energy efficiency. The main contributions of this work are as follows:

1. IFWH incorporates several strategies to improve trajectory optimization performance in complex simulated underwater environments. Piecewise linear chaotic mapping and balance operators improve obstacle avoidance and search-space coverage in offline simulations.
2. To handle path planning under ocean current constraints, IFWH integrates a logarithmic spiral local search strategy and quadratic interpolation, which generate smooth paths while respecting kinematic constraints and evaluating estimated propulsion energy cost features at the simulation level.
3. The offline simulation environment is progressively constructed from simple to complex scenarios to evaluate the performance of IFWH in simulations, considering terrain adaptation, obstacle avoidance, and propulsion energy cost estimation.

The remainder of this paper is organized as follows: Section 2 presents the mathematical model for simulation-based AUV path planning in submarine terrain. Section 3 introduces the basic FWH algorithm, and Section 4 describes the proposed IFWH algorithm. Section 5 presents offline MATLAB R2021a simulation experiments under representative terrain, obstacle, and ocean-current conditions to evaluate the path-planning performance of IFWH. Section 6 provides the conclusions and discusses the limitations of the study.

## 2. Mathematical Model for Dynamic AUV Path Planning in Coral Reef Areas

Coral reef ecosystems are highly complex and fragile, making AUV path planning in these scenarios a challenging multi-constraint optimization problem. Steep seabed terrain, complex coral formations, irregular topography, and moving obstacles can affect the feasibility and safety of the path. Therefore, appropriate mathematical models are needed to evaluate terrain constraints, obstacle threats, and path quality in simulation-based planning.

This study considers two main objectives: energy efficiency and navigation safety at the path-planning level. At the path-planning level, the estimated propulsion energy cost is affected by travel distance, current disturbance, relative velocity, and maneuvering behavior. Therefore, an energy-consumption cost function is introduced to evaluate the efficiency of the route in offline simulations.

Navigation safety is evaluated by considering the proximity of obstacles, the risk of collisions with terrain and maneuverability constraints. Path segments close to steep terrain, reef structures, or moving obstacles can increase the risk of collision in the planned trajectory. Excessive variations in slope-angle or turning-angle may also reduce the smoothness and feasibility of the path. Accordingly, this study constructs a unified multi-constraint cost function for simulation-based AUV path planning, including energy-consumption cost, obstacle-threat cost, terrain-collision risk cost, and maneuverability cost. It should be noted that the AUV model in this study is established to evaluate the trajectory-level and estimation propulsion costs during offline optimization. It is not intended to perform closed-loop dynamic simulation or onboard motion control.

According to the motion characteristics of the AUV, ignoring roll dynamics and AUV path planning is based on the kinematic and dynamic models of five-degrees-of-freedom [26]:

(1) $$\begin{array}{cc} \dot{x} & =u\cos(\theta )\cos(\psi )-v\sin(\psi )+w\sin(\theta )\cos(\psi )\\ \dot{y} & =u\cos(\theta )\sin(\psi )+v\cos(\psi )+w\sin(\theta )\sin(\psi )\\ \dot{z} & =-u\sin(\theta )+w\cos(\theta )\\ \dot{\theta } & =q\\ \dot{\psi } & =r\cos(\theta ) \end{array}$$

where *x*, *y*, and *z* denote the AUV position in the earth-fixed frame, while θ and ψ denote the pitch angle and yaw angle, respectively. The variables *q* and *r* denote the pitch and yaw angular velocities in the body-fixed frame, respectively.

(2) $$\begin{array}{cc} \dot{u} & =\frac{m_{22}}{m_{11}}vr-\frac{m_{33}}{m_{11}}wq-\frac{d_{11}}{m_{11}}u+\frac{1}{m_{11}}\tau_{u}\\ \dot{v} & =-\frac{m_{11}}{m_{22}}ur-\frac{d_{22}}{m_{22}}u\\ \dot{w} & =\frac{m_{11}}{m_{33}}uq-\frac{d_{33}}{m_{33}}w\\ \dot{q} & =\frac{m_{33}-m_{11}}{m_{55}}uw-\frac{d_{55}}{m_{55}}q-\frac{\rho g\nabla GM_{L}\sin\left(\theta \right)}{m_{55}}+\frac{1}{m_{55}}\tau_{q},\\ \dot{r} & =\frac{m_{11}-m_{22}}{m_{66}}uv-\frac{d_{66}}{m_{66}}r+\frac{1}{m_{66}}\tau_{r} \end{array}$$

where *u*, *v*, *w*, *q* and *r* are the components of the AUV velocity vector in the body-fixed reference frame, representing longitudinal, lateral, vertical linear velocities, and pitch, yaw angular velocities, respectively. $\tau_{u}$, $\tau_{q}$ and $\tau_{r}$ constitute the control input vector, corresponding to the thrust forces and moments exerted by the AUV thrusters. Parameters $m_{ii}\;\left(i=1,2,3,5,6\right)$ account for both rigid-body inertia and added hydrodynamic mass effects, while parameters $d_{ii}\;\left(i=1,2,3,5,6\right)$ characterize linear and nonlinear hydrodynamic damping forces. Furthermore, ∇ denotes the displacement volume of the AUV, *g* is the gravitational acceleration constant, ρ is the density of surrounding seawater, *G* is the total weight of the AUV, and $M_{L}$ is the vertical separation between the center of gravity (CG) and the center of buoyancy (CB), which determines the hydrostatic stability of the vehicle [27].

Equations (1) and (2) provide the physical basis for maneuverability at the planning-level and the evaluation of energy. They are not numerically integrated for every candidate path during IFWH optimization. Instead, their principal motion components are mapped to quantities that can be calculated directly from the waypoint sequence.

The surge component determines the commanded cruising speed used in the travel-time and propulsion-energy estimation. The heave and pitch components are reflected by vertical displacement and path-slope terms, while the yaw component is represented by the heading variation between adjacent path segments. Consequently, the proposed method is a dynamically informed and kinematically constrained geometric path planner rather than a complete actuator-level dynamic trajectory optimizer.

Let $Path_{i}$ represent the current path node of the AUV in the earth-fixed frame. Its next position is calculated and updated by (4):

(3) $$Path_{i}=\left(x_{i},y_{i},z_{i}\right)$$

(4) $$\begin{array}{cc} \dot{x} & =u\cos\theta \cos\psi -v\sin\psi +w\sin\theta \cos\psi \\ \dot{y} & =u\cos\theta \sin\psi +v\cos\psi +w\sin\theta \sin\psi \\ \dot{z} & =-u\sin\theta +w\cos\theta \end{array}$$

Based on the above model, the planner evaluates candidate waypoint sequences using terrain, obstacle, maneuverability, and energy-related quantities. The optimizer does not directly propagate the complete AUV dynamic state using Equations (1) and (2). Instead, it searches for a three-dimensional reference path that is compatible with the principal maneuvering characteristics of the AUV.

During optimization, each candidate path uses a fixed-dimensional representation. Let $N_{\max}$ denote the fixed number of optimized intermediate waypoints. The encoded path of individual *i* is defined as

(5) $$P_{i}^{\mathrm{enc}}=\left\{p_{s},p_{i,1},p_{i,2},\ldots ,p_{i,N_{\max}},p_{g}\right\},$$

where $p_{s}$ and $p_{g}$ are the prescribed start and goal positions and remain fixed during optimization, and

(6) $$p_{i,k}={\left[x_{i,k},y_{i,k},z_{i,k}\right]}^{T}$$

is the *k*th optimized intermediate waypoint.

The number of encoded intermediate waypoints remains fixed throughout the IFWH iterations. After optimization, redundant intermediate waypoints may be removed to obtain a more compact final path:

(7) $$P_{i}^{\mathrm{final}}=\left\{p_{s},p_{i,k_{1}},p_{i,k_{2}},\ldots ,p_{i,k_{N_{\mathrm{eff}}}},p_{g}\right\},\;N_{\mathrm{eff}}\leq N_{\max}.$$

Therefore, the variable number of waypoints refers only to the final post-processed path. The representation used by the histogram-based optimizer remains fixed-dimensional.

### 2.1. Propulsion Energy Cost Estimation Based on Thruster Characteristics

In simulation-based AUV path planning for submarine ecological monitoring scenarios, propulsion energy cost estimation is an important evaluation factor for simulation-based AUV path planning. The estimated propulsion energy cost is not only related to path length but also affected by local current disturbance, propulsion direction, attitude variation, and thruster efficiency. Therefore, using only the shortest path as the planning criterion may produce trajectories with higher estimated energy cost in counter-current or high-resistance regions. To evaluate this effect, an energy-consumption function based on hydrodynamic resistance and thruster characteristics is introduced into the path-planning fitness function.

The Euclidean distance between two adjacent nodes $p_{k}$ and $p_{k+1}$ is denoted as $d_{k}$:

(8) $$d_{k}={∥p_{k+1}-p_{k}∥}_{2}=\sqrt{{\left(x_{k+1}-x_{k}\right)}^{2}+{\left(y_{k+1}-y_{k}\right)}^{2}+{\left(z_{k+1}-z_{k}\right)}^{2}}.$$

In the *i*-th segment of the AUV path, the reference relative velocity of AUV to water is denoted as $V_{\mathrm{rel},i}$. The water resistance overcome by the AUV along a certain propulsion direction is denoted as $F_{Z,i}$:

(9) $$\begin{array}{cc} {\vec{V}}_{\mathrm{rel},i} & ={\vec{V}}_{\mathrm{set},i}-{\vec{V}}_{c,i}\\ V_{\mathrm{rel},i} & =∥{\vec{V}}_{\mathrm{rel},i}∥ \end{array}$$

where ${\vec{V}}_{\mathrm{set},i}$ represents the actual navigation speed of the AUV to the ground as set, and ${\vec{V}}_{c,i}$ represents the submarine current speed.

(10) $$F_{Z,i}=\frac{1}{2}C_{i}\rho S_{i}V_{\mathrm{rel},i}^{2}$$

where $C_{i}$ is the hydrodynamic coefficient of seawater, $S_{i}$ is the cross-sectional area of the AUV under force, ρ is the density of seawater. $C_{i}$ and $S_{i}$ can vary with the motion state and heading.

**Figure 1:**
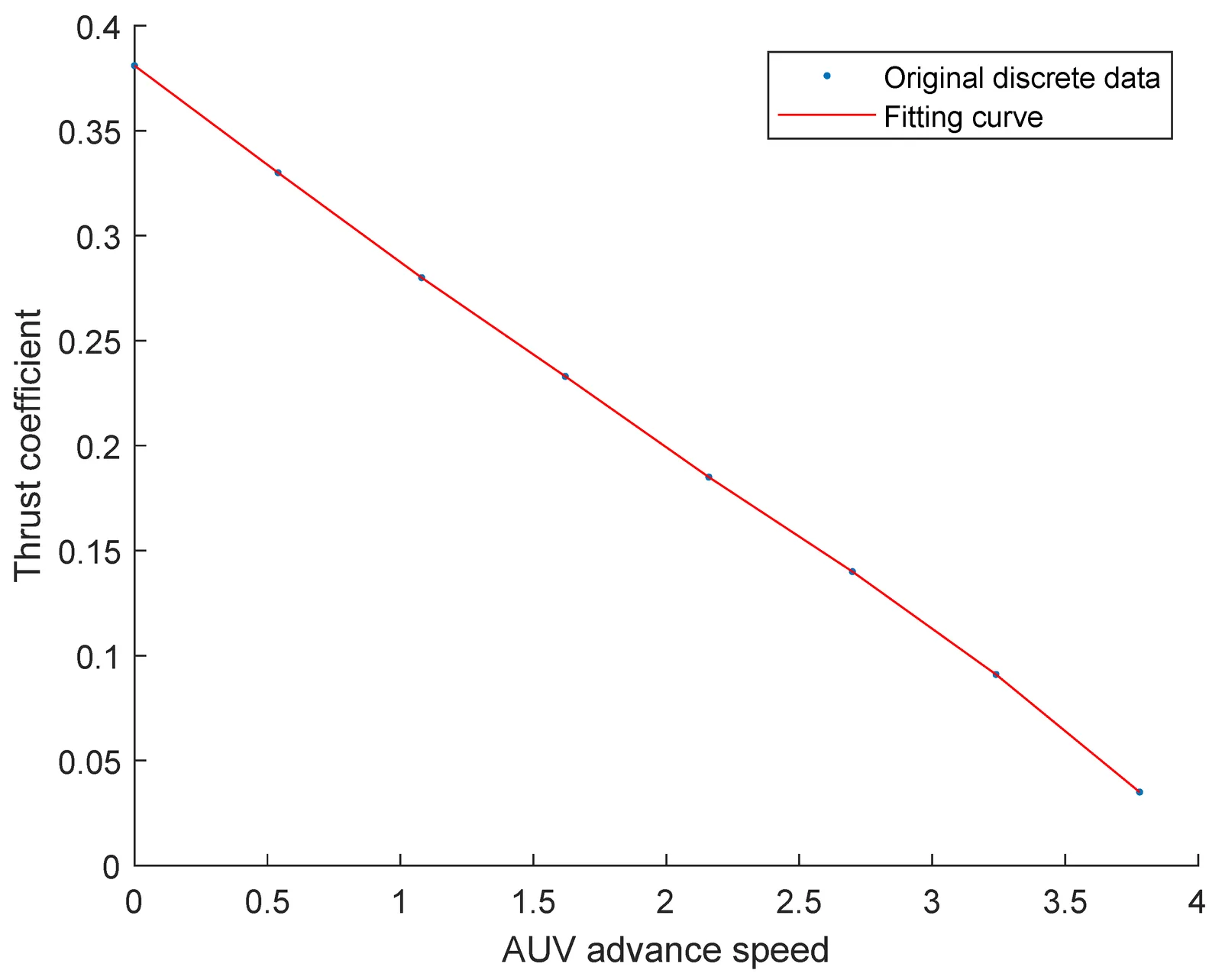
Relationship between thrust coefficient and advance speed.

**Figure 2:**
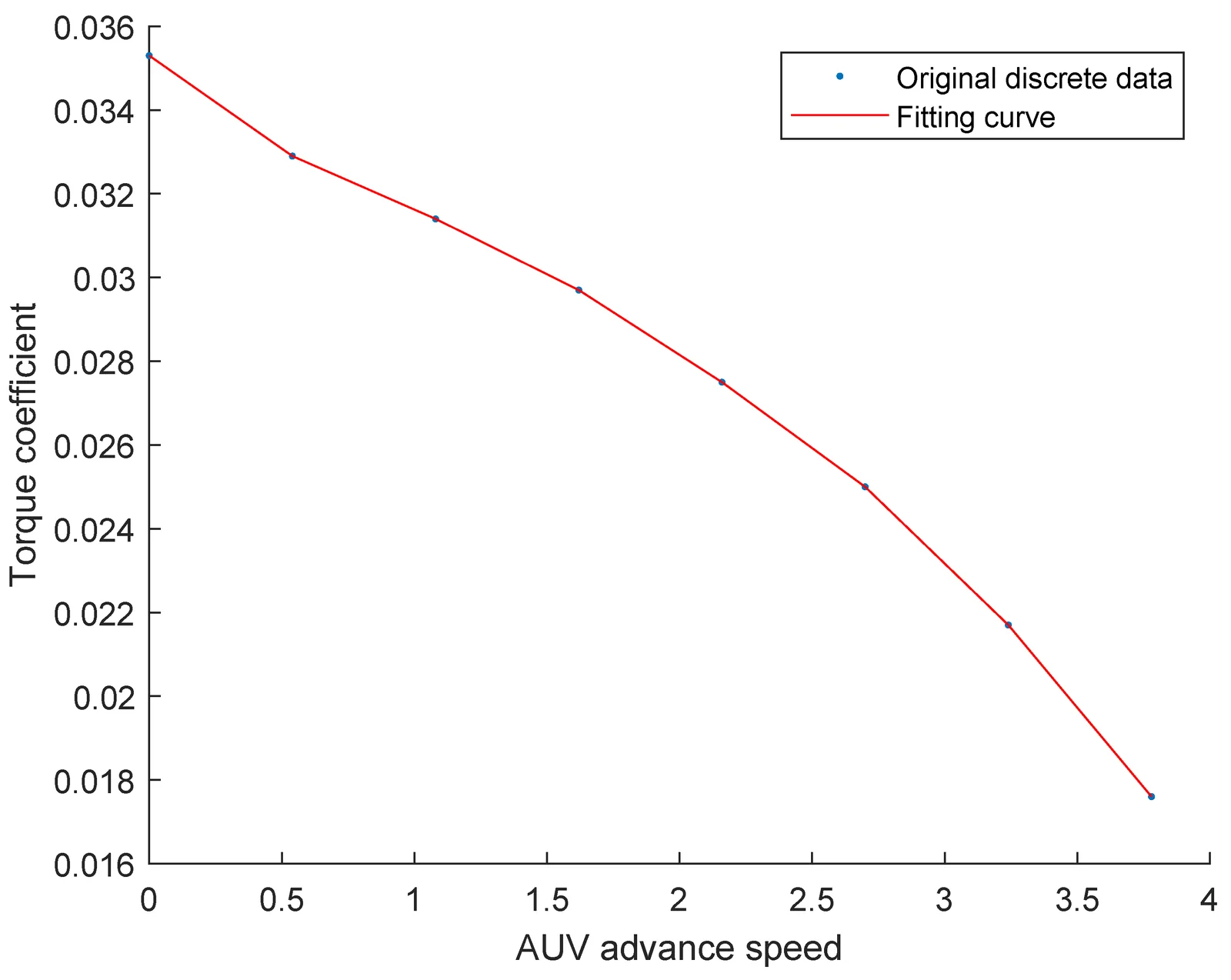
Relationship between torque coefficient and advance speed.

**Figure 3:**
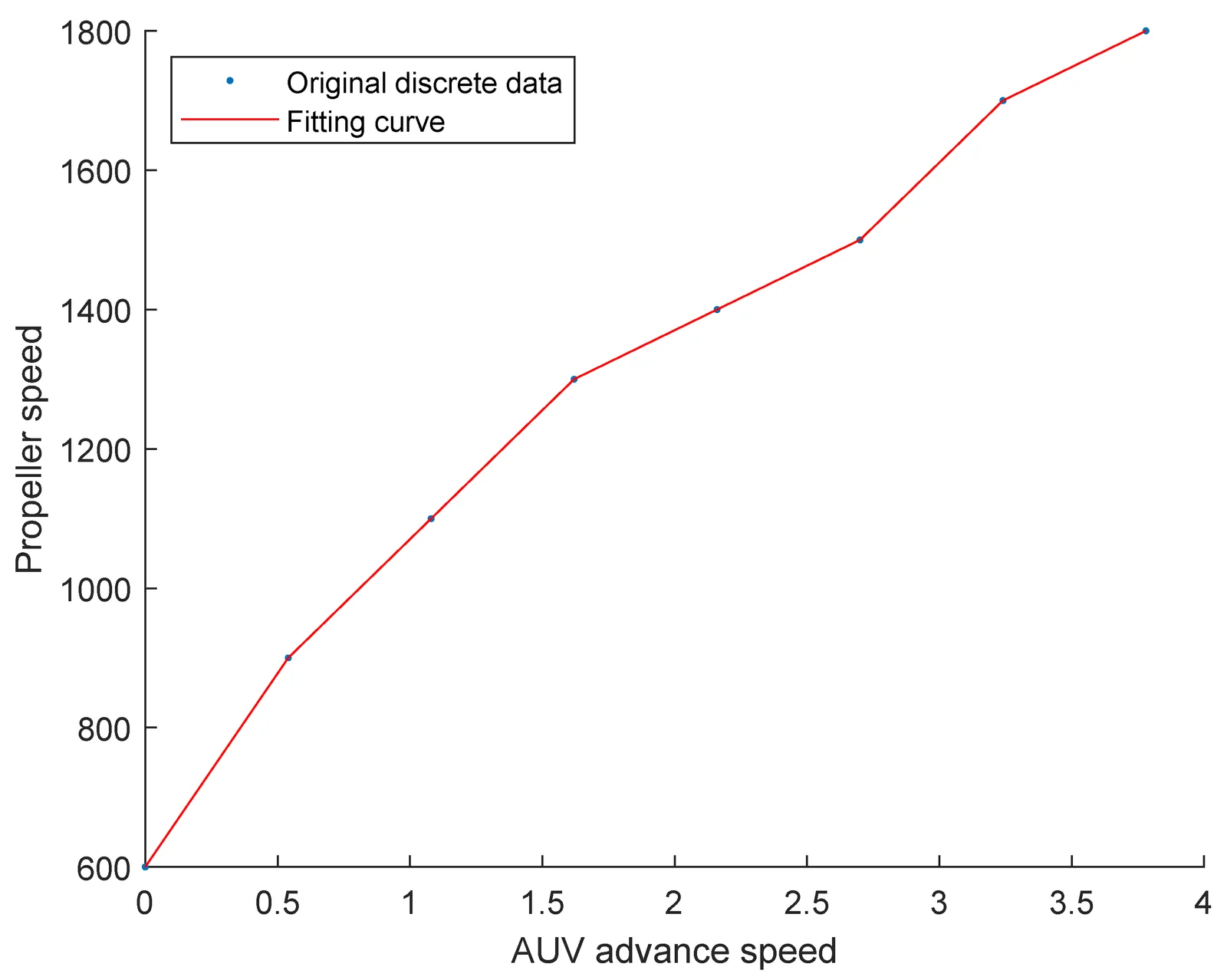
Relationship between propeller speed and advance speed.

In this paper, the formula for the efficiency of the AUV thruster adopted is

(11) $$\eta_{\mathrm{thrust},i}=\frac{K_{T}\left(V_{\mathrm{rel},i}\right)}{K_{Q}\left(V_{\mathrm{rel},i}\right)}\;\cdot \;\frac{V_{\mathrm{rel},i}}{2\pi n\left(V_{\mathrm{rel},i}\right)D}$$

where $K_{T}$ represents the thrust coefficient, $K_{Q}$ represents the torque coefficient, *n* represents the rotational speed of the propeller, and *D* represents the diameter of the propeller. $K_{T}$, $K_{Q}$ and *n* are obtained through multiple propulsion tests and are used to establish an approximate thruster-performance relationship within the tested operating range; the corresponding fitting curve is shown in Figure 1, Figure 2 and Figure 3.

(12) $$\begin{array}{cc} K_{T} & =-3.4702\times 10^{-4}V_{\mathrm{rel},i}^{2}-0.0887V_{\mathrm{rel},i}+0.3789\\ K_{Q} & =-6.1851\times 10^{-4}V_{\mathrm{rel},i}^{2}-0.0021V_{\mathrm{rel},i}+0.0348\\ n & =-38.7844V_{\mathrm{rel},i}^{2}+448.6332V_{\mathrm{rel},i}+637.5 \end{array}$$

The mechanical power required by the thruster to overcome this resistance is

(13) $$P_{i}=F_{Z,i}V_{\mathrm{rel},i}$$

After considering the efficiency of the thruster $\eta_{\mathrm{thrust},i}$, its propulsion energy cost is

(14) $$E_{i}=\frac{P_{i}t_{i}}{\eta_{\mathrm{thrust},i}}=\frac{F_{Z,i}V_{\mathrm{rel},i}t_{i}}{\eta_{\mathrm{thrust},i}}=\frac{F_{Z,i}d_{i}}{\eta_{\mathrm{thrust},i}}$$

In the *i*-th segment of the AUV path, if the AUV is propelled in the main, side and vertical directions respectively by thrusters in three directions, then the total propulsion energy cost is denoted as $E_{i}^{\mathrm{total}}$:

(15) $$E_{i}^{\mathrm{total}}=\frac{F_{Z,i}^{\left(M\right)}L_{i}^{\left(M\right)}}{\eta_{\mathrm{thrust},i}^{\left(M\right)}}+\frac{F_{Z,i}^{\left(H\right)}L_{i}^{\left(H\right)}}{\eta_{\mathrm{thrust},i}^{\left(H\right)}}+\frac{F_{Z,i}^{\left(V\right)}L_{i}^{\left(V\right)}}{\eta_{\mathrm{thrust},i}^{\left(V\right)}}$$

where $L_{i}^{\left(M\right)}$, $L_{i}^{\left(H\right)}$ and $L_{i}^{\left(V\right)}$ are the corresponding projection distances in the main, side, and vertical directions.

The total distance between nodes is taken as the principal metric, with the principal push taking the dominant position. Let $L_{i}^{\left(M\right)}\approx d_{i}$, and the lateral and vertical components are calculated based on the heading and pitch projections:

(16) $$\begin{array}{cc} L_{i}^{\left(M\right)} & =d_{i}\cos\theta_{i}\cos\psi_{i}\\ L_{i}^{\left(H\right)} & =d_{i}\cos\theta_{i}\sin\psi_{i}\\ L_{i}^{\left(V\right)} & =d_{i}\sin\theta_{i} \end{array}$$

The total propulsion energy cost of the thruster is obtained by accumulating the propulsion energy cost of each section:

(17) $$E_{\mathrm{prop}}=\sum_{i=0}^{N-1}E_{i}^{\mathrm{total}}=\sum_{i=0}^{N-1}\left[\frac{F_{Z,i}^{\left(M\right)}L_{i}^{\left(M\right)}}{\eta_{\mathrm{thrust},i}^{\left(M\right)}}+\frac{F_{Z,i}^{\left(H\right)}L_{i}^{\left(H\right)}}{\eta_{\mathrm{thrust},i}^{\left(H\right)}}+\frac{F_{Z,i}^{\left(V\right)}L_{i}^{\left(V\right)}}{\eta_{\mathrm{thrust},i}^{\left(V\right)}}\right]$$

It should be noted that the proposed energy model is designed for path-planning optimization rather than accurate prediction of the onboard electrical propulsion energy cost. The model estimates the relative propulsion energy requirement of different candidate trajectories based on hydrodynamic resistance and thruster efficiency characteristics. Electrical losses of the motor driver, battery discharge characteristics, and auxiliary equipment power consumption are not considered because this study focuses on trajectory-level optimization.

The cost function $F_{1}$ is given by (18):

(18) $$F_{1}=w_{E}E_{\mathrm{prop}}+w_{R}\sum_{i=0}^{N-1}R_{i}+w_{S}N_{\mathrm{steps}}+\mathrm{Penalty}$$

where $R_{i}$ represents the remaining distance from the node to the target point, which is used to enhance the global directivity of the path; $N_{\mathrm{steps}}$ represents the number of path nodes, which is used to limit redundant inflection points. Penalty is used for the constraints of other major penalties.

### 2.2. Obstacle Threat Cost

In submarine path-planning scenarios, static and moving obstacles can affect the feasibility of the planned trajectory. To evaluate these effects in offline simulations, this study constructs an obstacle-threat cost function that incorporates static and moving obstacle threats into a unified path-evaluation framework.

In this model, *M* static obstacles and *K* moving obstacles are defined in the simulated environment. The dynamic obstacle model is designed as a simplified prediction model for offline path-planning evaluation. Static obstacles are represented as three-dimensional fixed entities to describe coral colonies or rock masses. Moving obstacles are assigned preset initial positions, target positions, velocities, and movement directions. During each simulated path-update step, the possible next position of a moving obstacle is estimated according to its current position and preset movement direction. The threat of the obstacle to the current path segment is then evaluated based on the distance between the planned path and the obstacle.

The initial coordinates, current position and target endpoint of the *K*-th dynamic obstacle are expressed in sequence as ${\mathrm{Path}}_{k}^{\left(0\right)}$, ${\mathrm{Path}}_{k}\left(\tau \right)$, ${\mathrm{Path}}_{k}^{\left(g\right)}$.

Its motion update model is shown by (19).

(19) $$\begin{array}{cc} {\mathrm{Path}}_{k}\left(\tau +\Delta \tau \right) & ={\mathrm{Path}}_{k}\left(\tau \right)+v_{k}\Delta \tau \\ v_{k} & =∥v_{k}∥\\ \theta_{k} & =\arctan2\;\left({\mathrm{Path}}_{k,y}^{\left(g\right)}-{\mathrm{Path}}_{k,y}\left(\tau \right),{\mathrm{Path}}_{k,x}^{\left(g\right)}-{\mathrm{Path}}_{k,x}\left(\tau \right)\right)\\ v_{k} & =v_{k}\left[\cos\theta_{k},\;\sin\theta_{k}\right] \end{array}$$

where ${\mathrm{Path}}_{k}\left(\tau +\Delta \tau \right)$ is the position of the dynamic obstacle at the next moment; $v_{k}$ is the speed of the *K*-th dynamic obstacle; and $\theta_{k}$ represents the movement angle of the obstacle from its current position to its target direction.

To fully capture the three-dimensional impact of obstacles, the obstacle distances are extended to include vertical separation. For a dynamic obstacle, the 3D distance between the path node ${\mathrm{Path}}_{i}$ and obstacle *k* is

(20) $$D_{k}^{\left(3D\right)}=\sqrt{{\left(x_{i}-x_{k}\right)}^{2}+{\left(y_{i}-y_{k}\right)}^{2}+\lambda {\left(z_{i}-z_{k}\right)}^{2}}$$

where λ is a weighting factor for vertical separation, controlling its influence on the threat cost.

Similarly, for static obstacles,

(21) $$D_{m}^{\left(3D\right)}=\sqrt{{\left(x_{i}-x_{m}\right)}^{2}+{\left(y_{i}-y_{m}\right)}^{2}+\lambda {\left(z_{i}-z_{m}\right)}^{2}}$$

**Figure 4:**
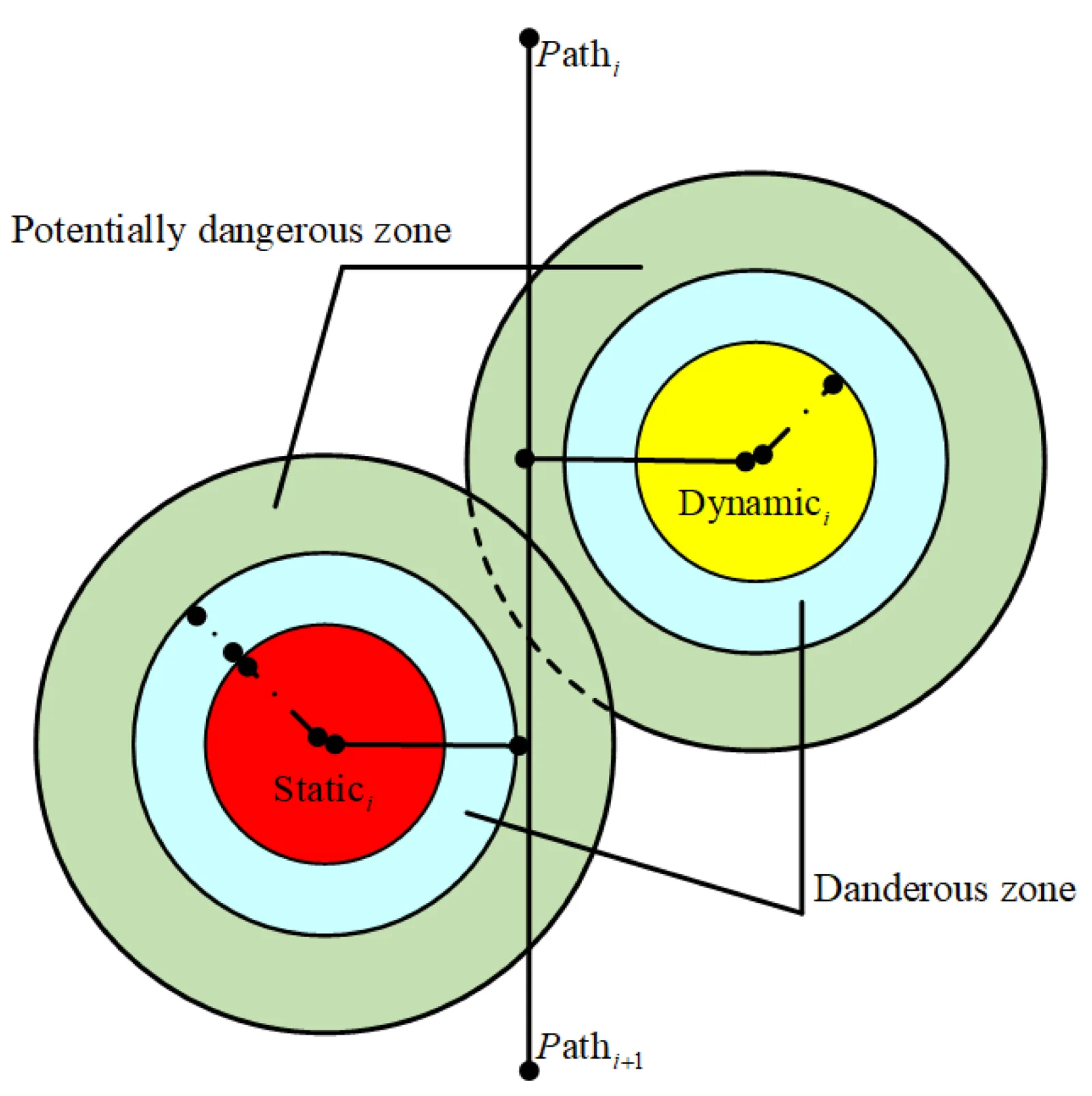
Obstacle threats to the navigation path.

When the AUV moves from the path node ${\mathrm{Path}}_{i}$ to the next node ${\mathrm{Path}}_{i+1}$, this path segment is threatened by nearby static and dynamic obstacles [28], as shown in Figure 4.

The red and yellow circular areas represent static and moving obstacles, respectively. The blue area denotes the obstacle influence range and the green area denotes the passable but potentially risky buffer zone for the planned path. Figure 4 provides a simplified schematic illustration of the obstacle-threat evaluation concept.

The three-dimensional obstacle-threat model is not established by the schematic itself but by the mathematical definitions of the distance and penalty functions. For both static and moving obstacles, the obstacle distance is evaluated in a three-dimensional space. For moving obstacles, the threat term also includes both the current obstacle position and its estimated next-step position. This design allows the cost function to evaluate the potential influence of moving obstacles on the planned path segment in offline simulations.

Based on the above analysis, the mathematical model $F_{2}$ of the obstacle threat cost function in the AUV path planning model is expressed by (22):

(22) $$F_{2}=\sum_{k=1}^{K}\left(T_{k}^{\mathrm{cur}}+T_{k}^{\mathrm{next}}\right)+\sum_{m=1}^{M}T_{m}^{\mathrm{static}}$$

The threat cost of dynamic obstacles consists of two parts—the threat brought by the current position at the moment and the threat brought by the predicted position at the next moment:

(23) $$\begin{array}{cc} T_{k}^{\mathrm{cur}} & =\left\{\begin{array}{cc} 0, & D_{k}^{\left(d\right)}\geq S_{k}^{\left(d\right)}\\ \alpha_{k}\left(S_{k}^{\left(d\right)}-D_{k}^{\left(d\right)}\right), & 0<D_{k}^{\left(d\right)}<S_{k}^{\left(d\right)}\\ +\infty , & D_{k}^{\left(d\right)}\leq 0 \end{array}\right.\\ T_{k}^{\mathrm{next}} & =\left\{\begin{array}{cc} 0, & D_{k}^{\left(d,\mathrm{next}\right)}\geq S_{k}^{\left(d\right)}\\ \beta_{k}\left(S_{k}^{\left(d\right)}-D_{k}^{\left(d,\mathrm{next}\right)}\right), & 0<D_{k}^{\left(d,\mathrm{next}\right)}<S_{k}^{\left(d\right)}\\ +\infty , & D_{k}^{\left(d,\mathrm{next}\right)}\leq 0 \end{array}\right. \end{array}$$

The cost of static obstacles is only related to the current distance:

(24) $$\begin{array}{cc} T_{m}^{\mathrm{static}} & =\left\{\begin{array}{cc} 0, & D_{m}^{\left(s\right)}\geq S_{m}^{\left(s\right)}\\ \gamma_{m}\left(S_{m}^{\left(s\right)}-D_{m}^{\left(s\right)}\right), & 0<D_{m}^{\left(s\right)}<S_{m}^{\left(s\right)}\\ +\infty , & D_{m}^{\left(s\right)}\leq 0 \end{array}\right.\\ D_{m}^{\left(s\right)} & =\mathrm{dist}\left(A_{i}A_{i+1},C_{m}^{\left(s\right)}\right)-R_{m}^{\left(s\right)}-W \end{array}$$

By introducing the obstacle threat cost function $F_{2}$, the path planning algorithm based on evolutionary computation can evaluate in real time the distance changes between the current path segment of the AUV and the surrounding dynamic and static obstacles at each step of path update, thereby achieving a dynamic response to environmental threats.

It should be noted that the current dynamic obstacle model focuses on evaluating the feasibility of path planning under predefined motion patterns. The uncertainty caused by sensor noise, incomplete environmental perception, and communication delays is not explicitly considered. Therefore, this study does not claim to provide a complete closed-loop dynamic obstacle avoidance framework for practical AUV deployment.

### 2.3. Cost of Terrain Collision Threat

In simulated submarine coral reef monitoring scenarios, the environment is characterized by complex three-dimensional terrain, including coral reef protrusions, steep seamounts, and irregular reef structures. These terrain features may significantly affect the feasibility and quality of the planned trajectory. If terrain constraints are not properly considered, the generated path may intersect with seabed terrain or reef structures in the simulation. Therefore, a terrain-collision cost function is introduced to evaluate topographic safety and eliminate infeasible path segments during the path-planning process.

To more accurately determine whether the AUV track will collide with the submarine topography, this study introduces three-dimensional spline interpolation between two adjacent path nodes ${\mathrm{Path}}_{i}{\mathrm{Path}}_{i+1}$ [29]. The interpolation method can generate a denser set of interpolation points in each flight segment:

(25) $$C_{i}=\left\{c_{i1},c_{i2},\ldots ,c_{iQ}\right\}$$

It contains *Q* intermediate points. The number of interpolation points *Q* is determined based on the complexity of the seabed topography and the resolution of the map.

Interpolation point $c_{iq}\left(x_{iq},y_{iq},z_{iq}\right)$ represents the coordinates of this point in three-dimensional space, where $z_{iq}$ indicates the depth of the AUV track at this point. The seabed elevation at the horizontal coordinate $\left(x_{iq},y_{iq}\right)$ is expressed as $H(x_{iq},y_{iq})$. By comparing the depth $z_{iq}$ of the interpolation point with the terrain height $H(x_{iq},y_{iq})$ of the corresponding position, if $z_{iq}$ is less than or equal to the terrain height, it indicates that the AUV track has collided with the seabed terrain or coral reef structure.

Based on the above definition, the terrain collision cost function $F_{3}$ of the AUV is expressed by (26):

(26) $$F_{3}\left(\mathrm{Path}\right)=\sum_{i=1}^{N}{\mathrm{Cost}}_{i}$$

The collision cost of each path segment is

(27) $${\mathrm{Cost}}_{i}=\sum_{q=1}^{Q}H_{iq}$$

The collision penalty term is defined as

(28) $$H_{iq}=\left\{\begin{array}{cc} \infty , & \mathrm{if}\;z_{iq}\leq H\left(x_{iq},y_{iq}\right)\\ 0, & \mathrm{else} \end{array}\right.$$

When a terrain collision occurs at any interpolation point, the cost is assigned to infinity to ensure that the path is completely eliminated during the optimization process, thereby guaranteeing that the AUV’s track does not have any physical collisions with coral reefs or submarine topography.

### 2.4. Dynamics-Informed Heading- and Slope-Angle Constraint Cost

In simulation-based AUV path planning, heading- and slope-angle variations are important factors affecting path smoothness and maneuverability. From the perspective of the 5-DOF model, abrupt changes in heading and slope correspond to rapid yaw and pitch maneuvers, which may increase the required control effort and estimated propulsion energy cost. Such changes may also produce paths that are difficult to execute under practical kinematic constraints. Therefore, heading- and slope-angle variation costs are introduced to penalize abrupt horizontal-direction and depth changes.

Let the candidate path contain *S* segments. For the *i*th segment connecting $p_{i}={\left[x_{i},y_{i},z_{i}\right]}^{T}$ and $p_{i+1}={\left[x_{i+1},y_{i+1},z_{i+1}\right]}^{T}$, the heading angle is defined as

(29) $$\psi_{i}=atan2\left(y_{i+1}-y_{i},\;x_{i+1}-x_{i}\right).$$

To avoid the angular discontinuity at ±π, the heading variation between two consecutive segments is calculated as

(30) $$\Delta \psi_{i}=\left|atan2\left[\sin\left(\psi_{i+1}-\psi_{i}\right),\cos\left(\psi_{i+1}-\psi_{i}\right)\right]\right|.$$

The slope angle of the *i*th segment is defined as

(31) $$\theta_{i}=atan2\left(z_{i+1}-z_{i},\;\sqrt{{\left(x_{i+1}-x_{i}\right)}^{2}+{\left(y_{i+1}-y_{i}\right)}^{2}}\right),$$

and the slope-angle variation between two consecutive segments is

(32) $$\Delta \theta_{i}=\left|\theta_{i+1}-\theta_{i}\right|.$$

Excessive values of $\Delta \psi_{i}$ and $\Delta \theta_{i}$ indicate abrupt yaw and pitch maneuvers, respectively. Therefore, the maximum allowable heading- and slope-angle variations are denoted by $\Delta \psi_{\max}$ and $\Delta \theta_{\max}$. A quadratic penalty is imposed only when the corresponding variation exceeds its prescribed threshold. This formulation allows small attitude adjustments while suppressing abrupt maneuvers.

The maneuverability cost associated with two consecutive path segments is defined as

(33) $${\mathrm{Cost}}_{i}^{\left(4\right)}=w_{\psi }f_{\psi }\left(\Delta \psi_{i}\right)+w_{\theta }f_{\theta }\left(\Delta \theta_{i}\right),$$

where $w_{\psi }$ and $w_{\theta }$ are the weights assigned to the heading- and slope-angle variation penalties, respectively. The heading-angle variation penalty is

(34) $$f_{\psi }\left(\Delta \psi_{i}\right)=\left\{\begin{array}{cc} 0,\; & \Delta \psi_{i}\leq \Delta \psi_{\max},\\ {\left(\Delta \psi_{i}-\Delta \psi_{\max}\right)}^{2},\; & \Delta \psi_{i}>\Delta \psi_{\max}. \end{array}\right.$$

Similarly, the slope-angle variation penalty is

(35) $$f_{\theta }\left(\Delta \theta_{i}\right)=\left\{\begin{array}{cc} 0,\; & \Delta \theta_{i}\leq \Delta \theta_{\max},\\ {\left(\Delta \theta_{i}-\Delta \theta_{\max}\right)}^{2},\; & \Delta \theta_{i}>\Delta \theta_{\max}. \end{array}\right.$$

Because a path containing *S* segments has S−1 pairs of consecutive segments, the total dynamics-informed maneuverability cost is

(36) $$F_{4}=\sum_{i=1}^{S-1}\left[w_{\psi }f_{\psi }\left(\Delta \psi_{i}\right)+w_{\theta }f_{\theta }\left(\Delta \theta_{i}\right)\right].$$

This cost maps the yaw- and pitch-related maneuvering characteristics of the 5-DOF model to quantities that can be evaluated directly from the waypoint sequence. It improves the smoothness and kinematic executability of the planned path without requiring repeated numerical integration of the complete nonlinear AUV dynamic equations.

### 2.5. Total Cost Function

Path planning is an important research problem in simulated submarine coral reef monitoring scenarios. In complex three-dimensional seabed terrain and current-disturbance conditions, a planned path should reduce path length, avoid intersections with static obstacles such as coral reefs and seamounts, consider the influence of moving obstacles, and maintain sufficient clearance from seabed terrain. These factors increase the complexity of the path-planning problem in environments with a complex obstacle system and terrain. Therefore, this paper proposes a comprehensive cost function for simulation-based AUV path planning in coral reef monitoring scenarios:

(37) $$F_{\mathrm{total}}\left(\mathrm{Path}\right)=w_{1}{\bar{F}}_{1}+w_{2}{\bar{F}}_{2}+w_{3}{\bar{F}}_{3}+w_{4}{\bar{F}}_{4}$$

To avoid weight imbalance caused by different dimensions, a normalized sub-term ${\bar{F}}_{j}$ (j=1,…,4) is used in the formula, which is defined by (38):

(38) $${\tilde{F}}_{j}=\frac{F_{j}-F_{j}^{\min}}{F_{j}^{\max}-F_{j}^{\min}+\epsilon }$$

where $F_{j}^{\max}$ and $F_{j}^{\min}$ represent the upper and lower bounds of the sub-costs observed in the current search space or historical samples, respectively, and ϵ is a small constant to prevent division by zero.

## 3. Basic Optimization Algorithm

The fixed-width histogram (FWH) [30] is a type of evolutionary algorithm based on probabilistic models. Its core idea is to construct a probabilistic model by statistically analyzing the distribution of high-quality solutions in the variable search space and to generate new candidate solutions based on this probabilistic model. This method belongs to a type of marginal estimation of distribution (EDA) algorithm. Its mechanism is similar to that of traditional genetic algorithms, but in the process of generating new solutions, probabilistic modeling is adopted instead of crossover and mutation operations, thus achieving a more robust search process. This section will introduce the population initialization, model construction methods, sampling mechanisms, and the overall framework of FWH.

### 3.1. Candidate Solution Representation and Trajectory Encoding

Although the final AUV path may contain a variable number of effective waypoints, IFWH does not directly optimize a variable-dimensional vector. Each individual contains exactly $N_{\max}$ intermediate three-dimensional waypoints during optimization.

The *i*th individual is encoded as

(39) $$X_{i}=\left[x_{i,1},y_{i,1},z_{i,1},x_{i,2},y_{i,2},z_{i,2},\ldots ,x_{i,N_{\max}},y_{i,N_{\max}},z_{i,N_{\max}}\right].$$

The prescribed start and goal positions are fixed and are not included as random variables in the histogram model. Therefore, the dimension of the optimization vector is

(40) $$D=3N_{\max}.$$

All individuals retain this dimension throughout population initialization, histogram construction, sampling, and local refinement. Consequently, the *j*th marginal histogram always represents the same coordinate component of the same intermediate waypoint.

After the termination of IFWH, redundant intermediate waypoints are removed from the best candidate path. For waypoint $p_{i,k}$, the direct segment connecting $p_{i,k-1}$ and $p_{i,k+1}$ is examined. The waypoint is removed only if the new segment is collision-free, maintains the required terrain clearance, and satisfies the prescribed heading and slope constraints. This procedure is repeated until no additional waypoint can be removed.

### 3.2. Population Initialization

In the process of solving the optimization problem, FWH first randomly initializes a candidate solution population composed of $N_{p}$ individuals. Each individual is a *D*-dimensional trajectory vector generated according to the encoding strategy described in Section 3.1. Let the dimension of the optimization problem be *n* and the domain of the *j*-th variable be $\left[\min_{j},\max_{j}\right]$. The initial population is randomly generated using a uniform distribution, and its mathematical expression is as follows:

(41) $$X_{i}^{j}=min_{j}+r\times \left(max_{j}-min_{j}\right),\;r\in \left[0,1\right]$$

where $X_{i}^{j}$ is the *j*-th dimensional variable of the *i*-th individual. *r* is the random number above. $\min_{j}$ and $\max_{j}$ represent the upper and lower bounds of the search space.

### 3.3. Construction of Marginal Fixed-Width Histogram Model

#### 3.3.1. Marginal Model Description

In each iteration, FWH first selects a set of high-quality solution sets S(t) based on fitness. Subsequently, statistical modeling is conducted for the variables of each dimension in the set S(t), and marginal histograms are constructed for each dimension variable $x_{j}$.

In the proposed IFWH algorithm, each dimension variable corresponds to one coordinate component of a waypoint in the encoded AUV trajectory. According to the trajectory encoding strategy described above, each individual contains $N_{\max}$ waypoints, and each waypoint consists of three spatial coordinates (x,y,z). Therefore, the dimension of the optimization vector is $D=3N_{\max}$, and the histogram model contains *D* marginal histograms corresponding to all waypoint coordinates, namely,

$$H_{1}\left(x_{1}\right),\;H_{2}\left(y_{1}\right),\;H_{3}\left(z_{1}\right),\;\ldots ,\;H_{D}\left(z_{N_{\max}}\right)$$

Specifically, the domain $\left[\min_{j},\max_{j}\right]$ of variable $x_{j}$ is divided into *H* fixed-width intervals, with the interval index being $h_{j}$:

(42) $$h_{j}=0,1,\ldots ,H-1$$

**Figure 5:**
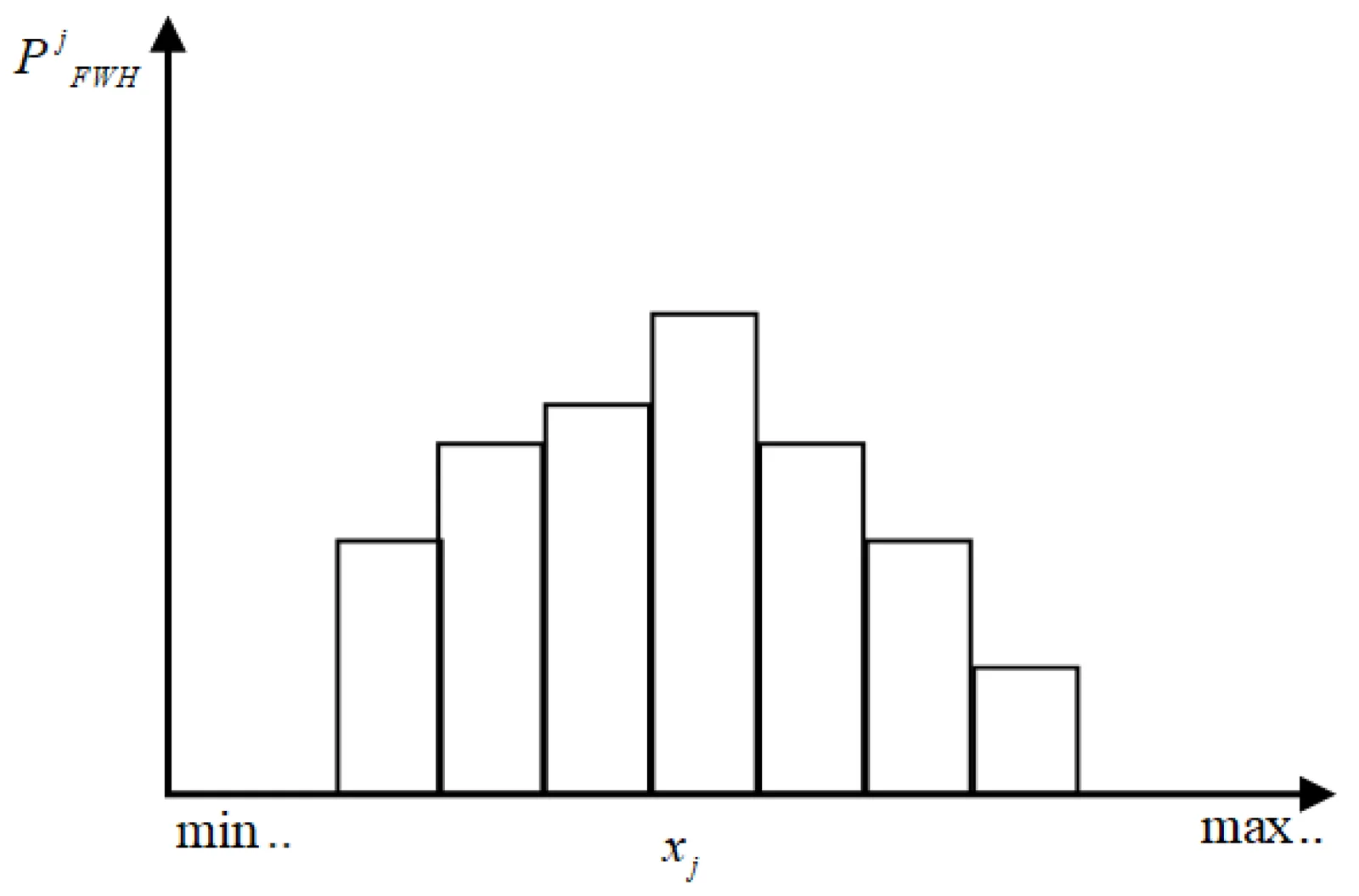
Marginal fixed-width histogram model.

The probability density estimation of the interval can be obtained based on the number of variable values falling into the interval in the high-quality solution:

(43) $$P_{FWH}^{j}\left[h\right]=\frac{∥\left\{V\left[i\right]\left[j\right]h\leq \frac{V\left[i\right]\left[j\right]-\min_{j}}{\max_{j}-\min_{j}}H<h+1\right\}∥}{N}$$

where V[i][j] represents the *j*-th variable of the *i*-th individual in the high-quality solution set, *N* is the population size, and $P_{FWH}^{j}\left[h\right]$ is the probability density of variable $x_{j}$ in the interval *h*. The entire model consists of *n* marginal histograms, with the total number of intervals in the histograms being nH. The structure of marginal fixed-width histogram is illustrated in Figure 5.

Since the marginal histogram model estimates each coordinate variable independently, the spatial dependency between consecutive waypoints is not explicitly represented in the probabilistic model. Instead, waypoint dependency is preserved through trajectory-level fitness evaluation.

Specifically, each sampled trajectory is evaluated using the integrated cost function, including path distance and propulsion energy cost, obstacle threat, terrain collision risk, and maneuverability constraints related to heading and slope angles. Therefore, infeasible combinations of adjacent waypoints will receive high penalty values and are gradually eliminated during the elite selection process.

#### 3.3.2. Interval Width Selection and Analysis of Multimodal Problems

The width of the interval is given by (44):

(44) $$\epsilon =\frac{\max_{j}-\min_{j}}{H}$$

**Figure 6:**
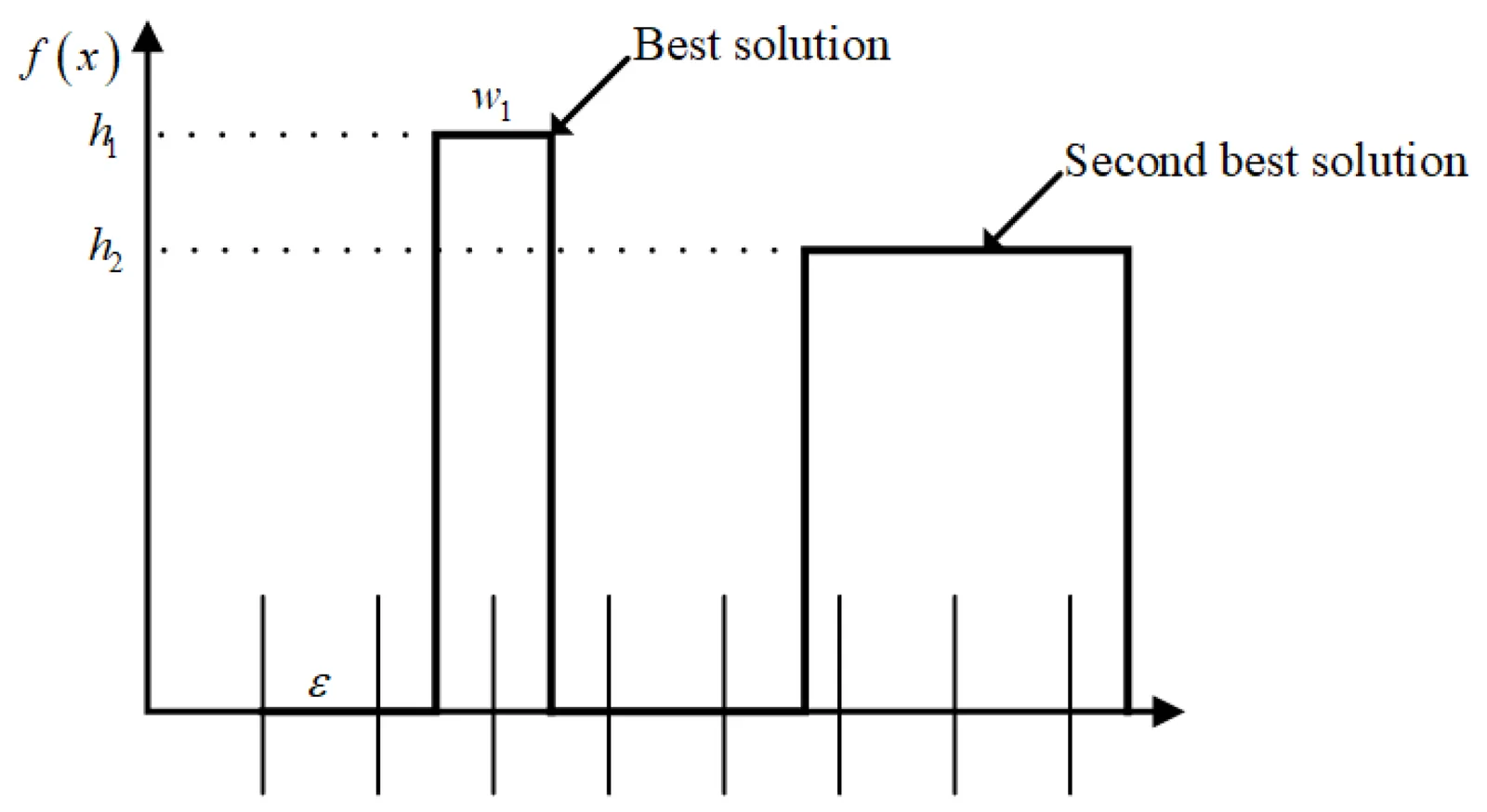
Multimodal fitness function.

For multi-modal optimization problems, if the interval width is too large, different peaks may be artificially combined, resulting in the density growth rate of the suboptimal peak being greater than that of the global optimal peak, causing erroneous convergence. To ensure that the model can identify the optimal global peak, (45) must be satisfied:

(45) $$\epsilon <\frac{v_{1}h_{1}}{2h_{2}}$$

where $w_{1}$ is the width of the optimal global peak. $h_{1}$ is the average fitness of the optimal peak. $h_{2}$ is the average fitness of the suboptimal peak.The multimodal fitness function is depicted in Figure 6.

### 3.4. Sampling Based on Histogram Model to Generate New Solutions

After constructing the histogram model, FWH uses it for sampling to generate a new solution set O(t). The sampling process consists of two steps: interval selection and uniform generation within the interval.

During each iteration, candidate solutions are ranked according to the total cost function. The top $N_{e}$ individuals are selected as elite solutions for histogram construction. The elite selection ratio is defined as

$$r_{e}=\frac{N_{e}}{N_{p}}$$

where $N_{p}$ denotes the population size and $N_{e}$ denotes the number of elite individuals.

The simplest method for interval selection is roulette (RW), but it introduces a relatively large sampling variance. Therefore, FWH adopts Extended Stochastic Universal Sampling (E-SUS) to reduce the sampling error. For each selected interval *h*, uniform random sampling is conducted within the corresponding interval $\left[L_{h},R_{h}\right]$:

(46) $$\begin{array}{cc} x_{j} & =L_{h}+r\times \left(R_{h}-L_{h}\right),\;r\in \left[0,1\right] \end{array}$$

(47) $$\begin{array}{cc} L_{h} & =min_{j}+h\;\cdot \;\epsilon ,\;R_{h}=L_{h}+\epsilon \end{array}$$

After sampling, the generated solution set O(t) is used as the population for the next iteration. The best solution is retained during the optimization process to maintain convergence stability.

## 4. The Proposed IFWH Algorithm for AUV Path Planning

Although the traditional FWH algorithm can rapidly estimate the probability distribution of different regions in complex environments, it still has deficiencies in the high-dimensional environment, the accuracy of the distribution approximation and the capture of local details. When the coral reef terrain shows obvious local sharp changes or dynamic obstacles frequently move, FWH is prone to problems such as smooth transitions or loss of details. To enhance the adaptability of FWH in complex seabed environments, this paper proposes an improved fixed-width histogram algorithm, termed IFWH algorithm. By integrating the chaotic sequence initialization strategy based on PWLCM, the quadratic interpolation strategy, the adaptive transfer strategy based on global search and local refinement, and the spiral search local enhancement strategy, IFWH can generate higher-quality planned paths in complex simulated submarine terrains, especially in terms of path feasibility, smoothness, obstacle avoidance, and propulsion energy cost estimation.

### 4.1. Chaotic Sequence Initialization Strategy Based on PWLCM

Traditional FWH usually generates sampling points randomly during the initialization stage. However, this approach not only fails to fully utilize prior sensor data, but also easily leads to uneven distribution and incomplete coverage, introducing statistical bias into the environmental model at an early stage. For this reason, this paper introduces the PWLCM chaotic sequence initialization strategy in the initial histogram construction stage of FWH. This strategy utilizes chaotic sequences to generate more sampling points in the passable areas identified by the sensors, while reducing the sampling density in obstacle areas. This method results in a more uniform distribution of initial samples throughout the environmental space.

The mathematical representation of the PWLCM chaotic map is (48):

(48) $$S_{\left(k+1\right)}=\left\{\begin{array}{cc} \frac{S_{k}}{r}, & 0\leq S_{k}<r\\ \frac{S_{k}-r}{0.5-r}, & r\leq S_{k}<0.5\\ \frac{1-r-S_{k}}{0.5-r}, & 0.5\leq S_{k}<1-r\\ \frac{1-S_{k}}{r}, & 1-r\leq S_{k}<1 \end{array}\right.$$

where *r* is the control parameter, s∈(0,1). A random sequence on the interval (0,1) is obtained through cyclic iteration, which has good statistical properties [31].

The generated chaotic sequence is mapped to the search interval $\left[\min_{j},\max_{j}\right]$ of each dimension to construct the initial sample points. If *N* initial samples need to be generated, then take the sequence value $s_{k(i,j)}$ for each sample *i* and dimension *j*, and the mapping formula is (49):

(49) $$X_{i,j}\left(0\right)=min_{j}+s_{k(i,j)}\;\cdot \;\left(max_{j}-min_{j}\right),\;i=1,\ldots ,N,\;j=1,\ldots ,n$$

Substitute the initial samples generated by PWLCM into a fixed-width histogram for statistics to obtain the initial frequency estimation of the *h*-th interval in the *j*-th dimension:

(50) $$P_{FWH}^{j}\left[h\right]=\frac{1}{N}∥\left\{X_{i,j}\left(0\right)|h\leq \frac{X_{i,j}\left(0\right)-\min_{j}}{\max_{j}-\min_{j}}H<h+1\right\}∥$$

To prevent the occurrence of zero-probability intervals, a smoothing constant η>0 is added to all $P_{FWH}^{j}\left[h\right]$ during implementation and normalized:

(51) $${\tilde{P}}_{FWH}^{j}\left[h\right]=\frac{P_{FWH}^{j}\left[h\right]+\eta }{\sum_{q=0}^{H-1}\left(P_{FWH}^{j}\left[q\right]+\eta \right)}$$

### 4.2. Quadratic Interpolation Strategy

Due to the fixed-width division method adopted by traditional FWH, there is a problem of insufficient resolution in detail expression, which makes it impossible to accurately capture local terrain changes and thus affects the smoothness and feasibility of the path. To solve this problem, this paper introduces the quadratic interpolation strategy. When the sensor detects local terrain undulations or small obstacles not included in the prior map, the algorithm triggers the quadratic interpolation module to generate denser local refinement points without changing the dimension of the encoded trajectory vector.

Three points $\left(s_{1},v_{1}\right)$, $\left(s_{2},v_{2}\right)$, $\left(s_{3},v_{3}\right)$ are selected within the current analysis range of a certain dimension *j*. $s_{i}$ is the independent variable of this dimension, and $v_{i}$ is the evaluation value at this position. Fit with a quadratic polynomial $p_{j}\left(s\right)$ in this dimension. The coefficients α,β,γ are determined by the linear system:

(52) $$\begin{array}{cc} p_{j}\left(s\right) & =\alpha s^{2}+\beta s+\gamma \end{array}$$

(53) $$\left[\begin{array}{ccc} s_{1}^{2} & s_{1} & 1\\ s_{2}^{2} & s_{2} & 1\\ s_{3}^{2} & s_{3} & 1 \end{array}\right]\left[\begin{array}{c} \alpha \\ \beta \\ \gamma \end{array}\right]\;=\left[\begin{array}{c} v_{1}\\ v_{2}\\ v_{3} \end{array}\right]$$

If α≠0, the extreme points of the quadratic function are determined by (54). If $s^{*}$ exceeds the boundary or α is approximately 0, the degradation strategy is to directly select the corresponding position with the best cost among the current three points, as shown in (55):

(54) $$\begin{array}{cc} s^{*} & =-\frac{\beta }{2\alpha } \end{array}$$

(55) $$\begin{array}{cc} s^{*} & \leftarrow \arg\min_{s\in \{s_{1},s_{2},s_{3}\}}v\left(s\right) \end{array}$$

### 4.3. Adaptive Transfer Strategy for Global Search and Local Refinement

In the histogram update stage, traditional FWH often fails to dynamically adjust the search strategy based on the complexity of the environment, resulting in the coexistence of global statistical bias and the lack of local details. To solve this problem, this paper designs an adaptive transfer strategy based on global search and local refinement. In the early stage of the search, IFWH models the macroenvironment based on the statistical trend of the global histogram, enhancing the exploration ability of the overall safety zone, the distribution of major obstacles and low-cost channels. In the later stage of the search, when the candidate paths gradually concentrate in several advantageous intervals, the algorithm automatically enhances the weight of local refinement, gradually shifting the update process towards high-precision environmental estimation.

Define a control parameter ω(t)∈[0,1] to represent the global preference degree. The larger ω(t) is, the more it leans towards global sampling, and it varies with the number of iterations *t*:

(56) $$\omega \left(t\right)=\omega_{\min}+\left(\omega_{\max}-\omega_{\min}\right)\left(1-{\left(\frac{t}{T}\right)}^{\kappa }\right),\;t=0,1,\ldots ,T$$

where *T* is the maximum number of iterations; $\omega_{\max}$ and $\omega_{\min}$ are the initial and final global preference weights, respectively; and κ>0 controls the attenuation rate.

The adaptive transfer rule based on ω(t) is as follows: when generating each new sample in the number of iterations *t*, a uniform random number u∼U(0,1) is first taken. If u<ω(t), IFWH performs global histogram sampling. Otherwise, IFWH performs local refinement, prioritizing the use of quadratic interpolation or spiral search to generate samples near the dominant interval.

### 4.4. Spiral Search Local Enhancement Strategy

In highly complex coral-dense areas, there may still be some critical path regions that require more in-depth local searches. For this reason, this paper further introduces the local enhancement strategy of spiral search. Spiral search utilizes the gradually contracting spiral trajectory to conduct multi-directional and multi-scale detection of the dominant interval, which can help the algorithm perform denser local estimation in key areas, thereby obtaining higher-accuracy environmental information.

Let the projection of the center of a certain dominant interval on the two-dimensional plane be $c=(c_{x},c_{y})$. Define the initial radius $r_{0}>0$, the contraction factor ρ∈(0,1) at each step, and the angular step size Δθ. Then, the spiral point at the *k*-th step is given by (57):

(57) $$\left\{\begin{array}{c} r_{k}=r_{0}\rho^{k},\\ \theta_{k}=\theta_{0}+k\Delta \theta ,\\ p_{k}=c+r_{k}\left[\begin{array}{c} \cos\theta_{k}\\ \sin\theta_{k} \end{array}\right] \end{array}\right.$$

where $k=0,1,\ldots ,K_{\max}$; $\theta_{0}$ is the starting angle. For an *n*-dimensional space, points can be generated within a local plane composed of two principal directions u,v using the same formula:

(58) $$p_{k}=c+r_{k}\left(\cos\theta_{k}\;u+\sin\theta_{k}\;v\right)$$

where *c* represents the position of the current advantageous range; u,v represents the two orthogonal directions of this point locally. Generate a series of $p_{k}$ points and map them back to the feasible region; evaluate the cost $f(p_{k})$ of each $p_{k}$ and add the better ones to the new candidate set.

### 4.5. The Proposed IFWH Algorithm

In the complex coral reef seabed terrain environment, the AUV path planning problem poses higher requirements for the algorithm’s global optimization ability, local search accuracy, and dynamic response speed. The general process of the path planning strategy based on the IFWH algorithm is shown in Algorithm 1.**Algorithm 1** IFWH planning and time-resolved collision validation**Require:** Start point $P_{s}$, goal point $P_{g}$, search space Ω, terrain map H(x,y), static obstacles $O_{s}$, dynamic obstacles $O_{d}$, ocean-current field $V_{c}$, population size *N*, maximum optimization iteration number *T*, environmental time step Δτ, and number of histogram intervals $H_{b}$.**Ensure:** Best feasible path $P^{*}$.  1:Initialize the AUV parameters, cost-function weights, safety thresholds, and IFWH parameters. Set the environmental time τ=0 and optimization iteration index i=0.  2:Generate the initial population $P^{\left(0\right)}=\left\{P_{1}^{\left(0\right)},P_{2}^{\left(0\right)},\ldots ,P_{N}^{\left(0\right)}\right\}$ using the PWLCM chaotic mapping strategy.  3:Update the dynamic environment according to the physical simulation time:$$O_{d}\left(\tau +\Delta \tau \right)=M\left(O_{d}\left(\tau \right)\right),$$
where M(·) denotes the dynamic obstacle motion model. Estimate the next-step positions of dynamic obstacles.  4:**while **i<T** do**  5:    Evaluate each candidate path $P_{j}^{\left(i\right)}\in P^{\left(i\right)}$ under the current environmental state at time τ. Check whether the candidate path satisfies the boundary constraint of search space Ω. If the candidate violates the constraint, assign a sufficiently large penalty.  6:    For each feasible candidate path, compute the propulsion-energy cost $F_{1}\left(P_{j}^{\left(i\right)}\right)$, obstacle-threat cost $F_{2}\left(P_{j}^{\left(i\right)}\right)$, terrain-collision cost $F_{3}\left(P_{j}^{\left(i\right)}\right)$, and maneuverability cost $F_{4}\left(P_{j}^{\left(i\right)}\right)$.  7:    **if** terrain collision occurs or the candidate path intersects an obstacle **then**  8:        Assign an infinite or sufficiently large penalty.  9:    **else**10:        Calculate the total cost:$$F_{\mathrm{total}}\left(P_{j}^{\left(i\right)}\right)=w_{1}{\bar{F}}_{1}+w_{2}{\bar{F}}_{2}+w_{3}{\bar{F}}_{3}+w_{4}{\bar{F}}_{4}.$$11:    **end if**12:    Select elite candidate paths according to $F_{\mathrm{total}}$ and update the fixed-width histogram probability model using the elite paths.13:    Compute the adaptive global-search weight ω(i). For each new candidate path, generate a random number u∼U(0,1).14:    **if** u<ω(i) **then**15:        Generate the candidate by global histogram sampling;16:    **else**17:        Perform local refinement using quadratic interpolation or spiral search.18:    **end if**19:    Form the new population $P^{\left(i+1\right)}$ using the newly generated candidates and elite retained paths, and update i←i+1.20:**end while**21:After the maximum optimization iteration number is reached, output the feasible path with the minimum total cost as $P^{*}$.22:When replanning is required, update the environmental time τ←τ+Δτ and repeat Steps 3–10 using the updated dynamic obstacle states.

### 4.6. Computational Complexity and Runtime Analysis

To avoid ambiguity, different symbols are used for the grid size and the population-related quantities. Let $V=N_{x}N_{y}$ denote the number of grid nodes, $N_{p}$ the population size, $D=3N_{\max}$ the dimension of an encoded candidate trajectory, *T* the maximum number of iterations, $H_{b}$ the number of histogram intervals per variable, *S* the number of path segments, *M* the number of obstacles, and $M_{d}$ the number of dynamic obstacles.

For A^*^, the grid contains *V* vertices and at most 8V edges because an eight-connected neighborhood is used. With a binary-heap priority queue, its complexity is

(59) $$O\left((V+E)\log V\right)=O\left(V\log V\right),$$

where E≤8V. In the current MATLAB implementation, the minimum open-set cost is obtained by scanning the grid. Therefore, its practical worst-case complexity is $O(V^{2})$.

Both FWH and IFWH are population-based estimation-of-distribution algorithms. For each iteration, evaluating the trajectory costs of all individuals requires approximately $O(N_{p}D)$. Elite selection requires $O(N_{p}\log N_{p})$ when sorting is used. Constructing the marginal histogram model requires $O(N_{p}D+DH_{b})$, while sampling a new population requires $O(N_{p}D)$.

The collision-evaluation cost depends on the obstacle representation. When occupancy is queried directly from a grid map, checking all segments of all candidate trajectories requires $O(N_{p}S)$. When every trajectory segment is explicitly checked against *M* individual obstacles, the worst-case collision checking cost becomes $O(N_{p}SM)$. Therefore, the total worst-case complexity of FWH over *T* iterations is

(60) $$C_{\mathrm{FWH}}=O\left[T\left(N_{p}D+N_{p}\log N_{p}+DH_{b}+N_{p}SM\right)\right].$$

IFWH retains the population evaluation, elite selection, histogram construction, and sampling operations of FWH. It additionally introduces chaotic population initialization, an adaptive global–local search strategy, quadratic interpolation, spiral refinement, and dynamic-obstacle prediction. Chaotic initialization is performed once and requires $O(N_{p}D)$. In the worst case, local refinement of the candidate population requires an additional $O(N_{p}D)$ per iteration, while updating and predicting $M_{d}$ dynamic obstacles requires $O(M_{d})$ per iteration. Thus, the total complexity of IFWH is

(61) $$\begin{array}{c} C_{\mathrm{IFWH}}=O\left(N_{p}D\right)+O\left[T\left(N_{p}D+N_{p}\log N_{p}+DH_{b}+N_{p}SM+M_{d}\right)\right]. \end{array}$$

Compared with the original FWH algorithm, IFWH therefore has a higher actual computational cost because of chaotic initialization, adaptive search control, local refinement, and dynamic-obstacle prediction. However, these additional operations are linear in the population size, trajectory dimension, or number of dynamic obstacles. They do not introduce a higher-order term than the population fitness evaluation and collision checking already required by FWH. Consequently, when $N_{p}$, *D*, *S*, and *M* dominate the computation, FWH and IFWH retain the same leading asymptotic complexity order. The improvement of IFWH is therefore achieved at the cost of a moderate increase in constant factors and lower-order computational terms rather than a change in the dominant Big-*O* order. The computational complexity of different algorithms is summarized in Table 1.

## 5. Simulation Experiments and Analysis

### 5.1. Simulation Experiment

To evaluate the effectiveness of the proposed IFWH algorithm at the simulation-based path-planning level, this paper establishes an offline MATLAB R2021a simulation environment with typical coral-reef seabed terrain features and conducts comparative experiments under different environmental complexities. Five representative path-planning algorithms are considered, including A* [32,33], the original FWH, IPSO, and SVF-RRT*.

All experiments were implemented in MATLAB R2021a and conducted under 64-bit Windows 11 on a laptop equipped with an Intel Core i7-14650HX processor, 16 GB DDR5 memory at 5600 MT/s, and an NVIDIA GeForce RTX 4060 Laptop GPU with 8 GB memory (Lenovo, Hong Kong, China). The GPU was not used in the present implementation. When available, a MATLAB R2021a local parallel pool was employed only to evaluate the fitness values of different population individuals in parallel. The reported planning time includes only the execution of the IFWH optimization and excludes environment generation, final trajectory validation, data export, and figure generation.

#### 5.1.1. Parameter Settings

The parameters of IFWH and the baseline methods were selected empirically through preliminary feasibility and convergence tests. The selection aimed to ensure stable convergence, a sufficiently high success rate, and the absence of obvious path oscillations. An exhaustive parameter search was not performed; therefore, the selected values are not claimed to be globally optimal.

After selection, all parameters were fixed and applied unchanged to both scenarios and all trials. No scenario-specific tuning was performed. The same map, start–goal configuration, grid resolution, parameters, and evaluation criteria were used for all methods. The final parameter settings are listed in Table 2.

#### 5.1.2. Simulation Experiment Under Static Obstacles

**Figure 7:**
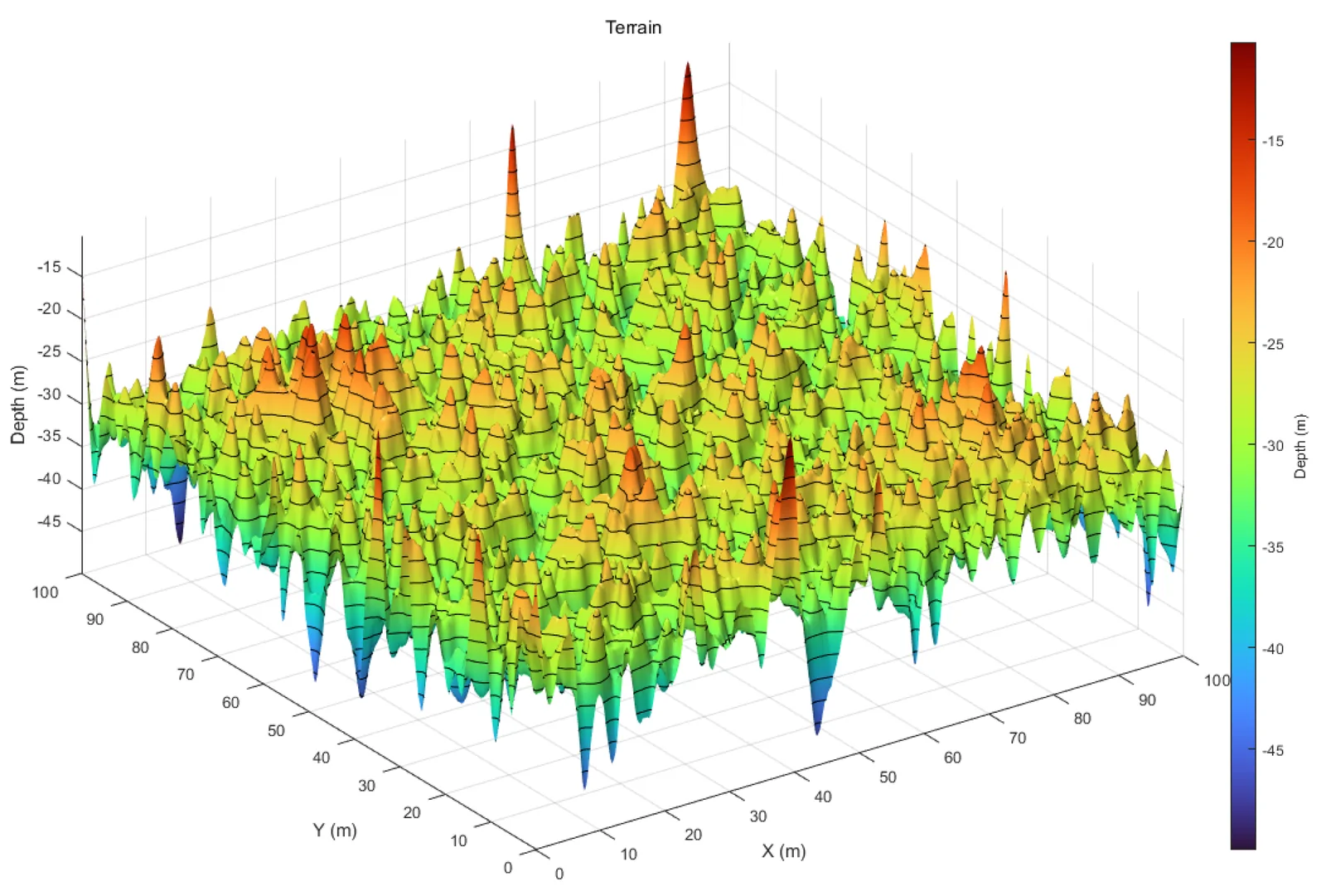
Elevation terrain of Scene 1.

Firstly, the experiment conducted basic performance tests in an environment that contained only static coral reef obstacles. According to the vertical variation amplitude of the obstacles, the experiment is divided into two sub-scenarios:

Scene 1: The height variation of the obstacles is relatively small, and the three-dimensional environment can be reduced to a two-dimensional planar path planning, which is used to verify the algorithm’s optimization ability under simple terrain constraints. The elevation terrain of Scene 1 is shown in Figure 7.

**Figure 8:**
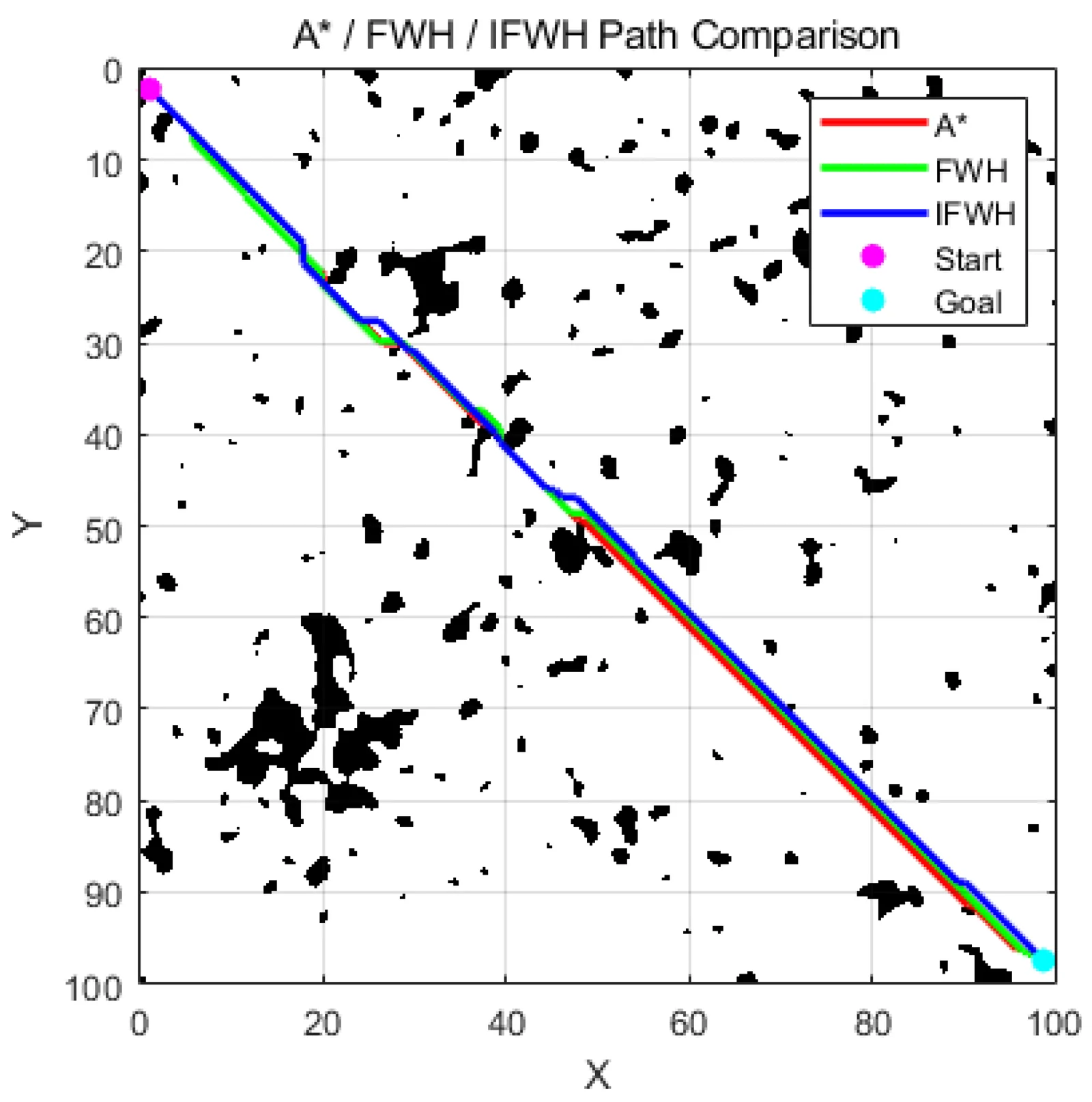
Extracted terrain map at a depth of 25 m.

**Figure 9:**
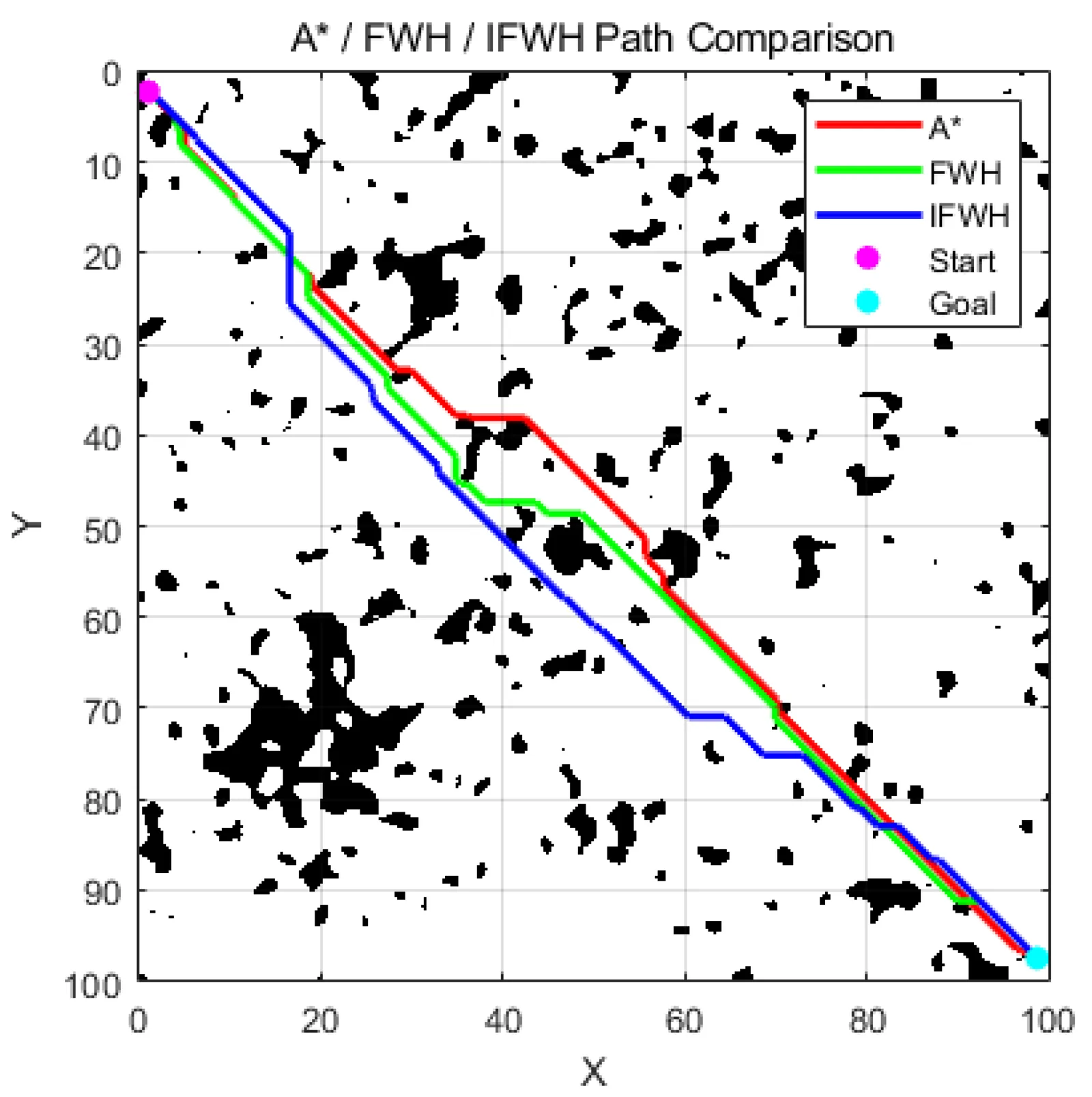
Extracted terrain map at a depth of 26 m.

**Figure 10:**
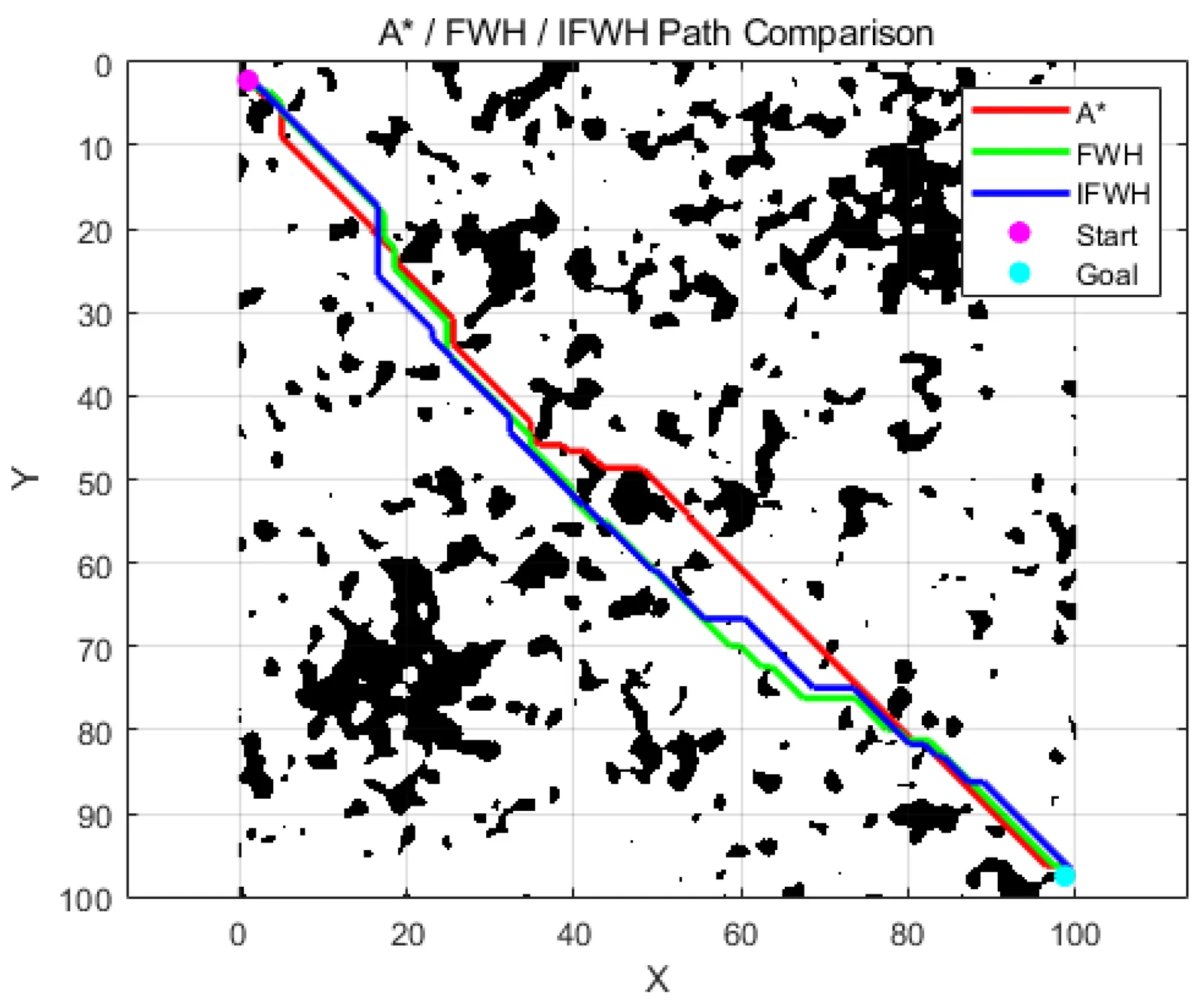
Extracted terrain map at a depth of 27 m.

**Figure 11:**
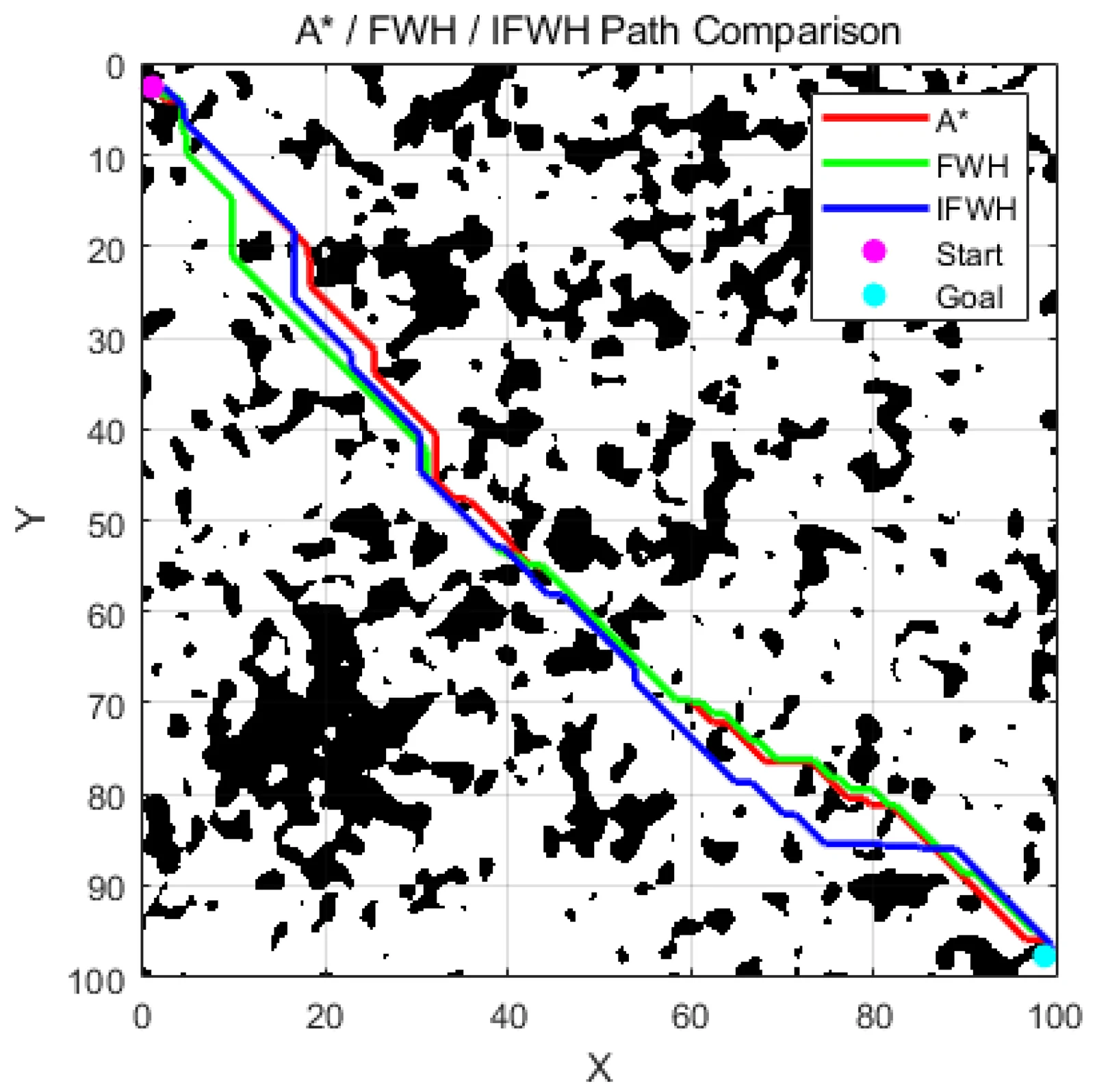
Extracted terrain map at a depth of 28 m.

The experiment first extracts two-dimensional maps with depths of 25 m, 26 m, 27 m, and 28 m from the elevation map, as illustrated in Figure 8, Figure 9, Figure 10, and Figure 11. Then the A*, FWH, and IFWH algorithms are used to conduct path planning under these four depth maps, respectively. Through experiments, it was found that when the environment is very simple, the paths planned by the three algorithms almost overlap. As the environment becomes increasingly complex, the path planned by the IFWH algorithm is clearly superior to that of other algorithms.

Scene 2: The obstacle has a significant height difference, and a feasible path must be planned in a three-dimensional space. In more complex simulation environments, simple two-dimensional planning is no longer applicable. The algorithms used need to perform operations such as ascending and descending to avoid obstacles.

**Figure 12:**
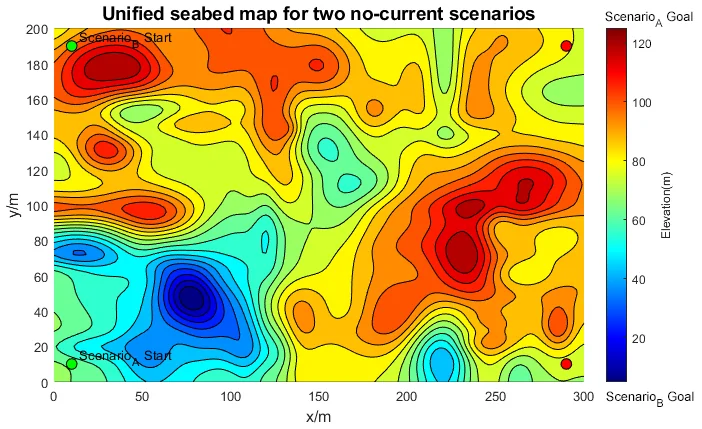
Unified seabed map for two current-free scenarios.

**Figure 13:**
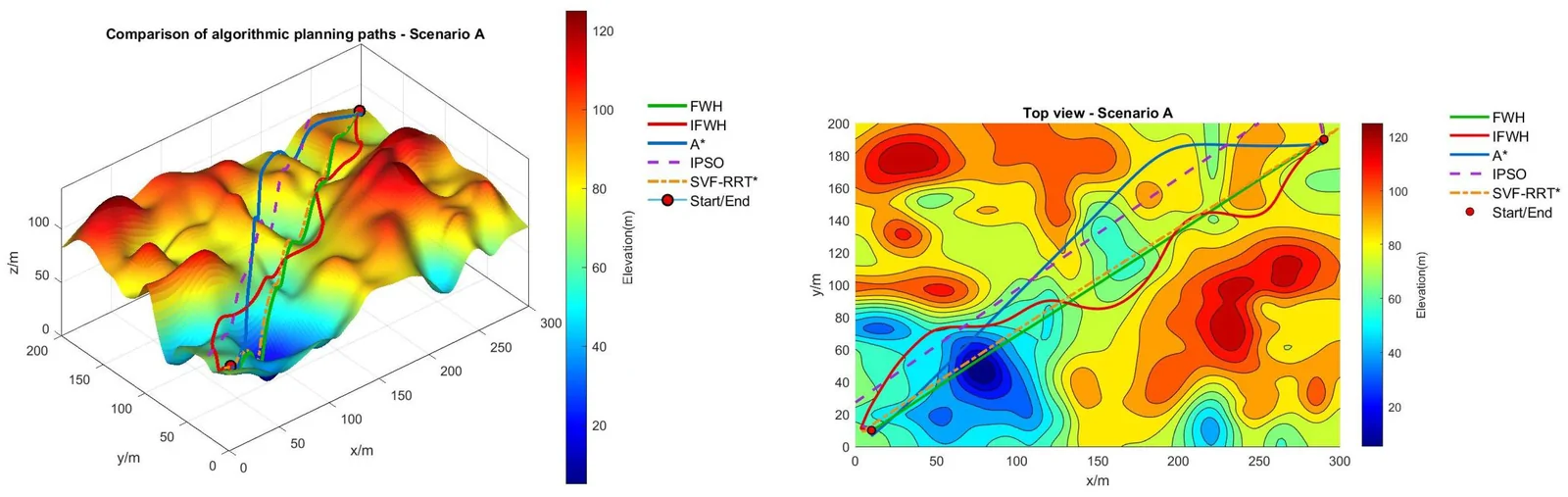
Comparison of algorithmic planning paths in Scenario A.

**Figure 14:**
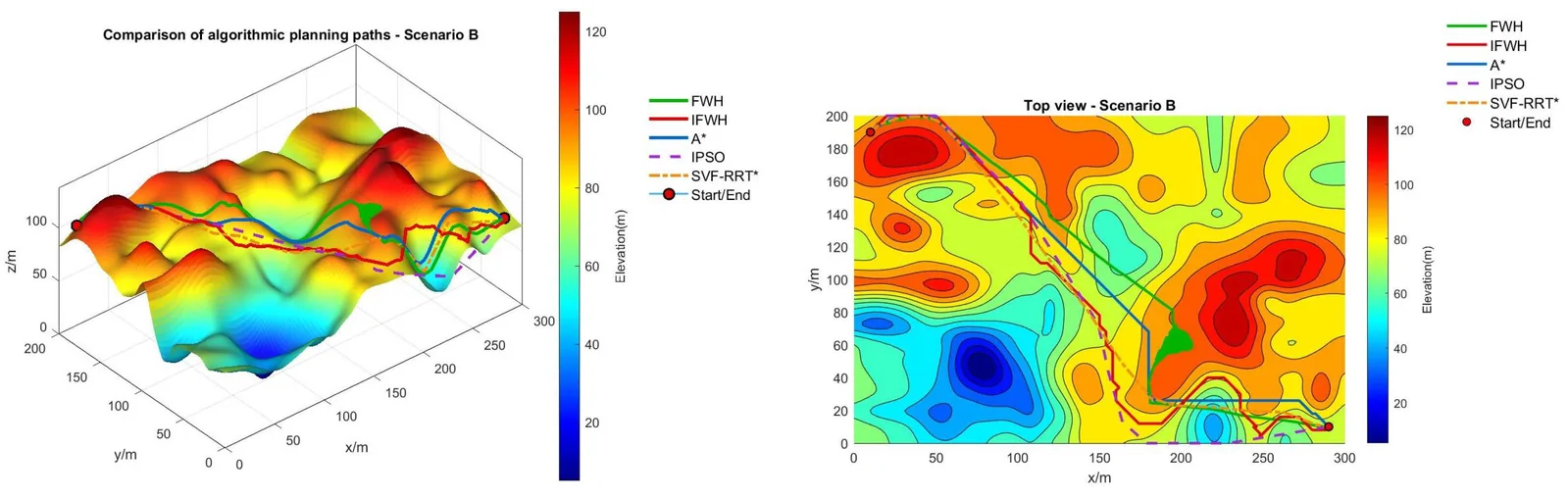
Comparison of algorithmic planning paths in Scenario B.

To further verify the terrain adaptability of the proposed IFWH algorithm, two path-planning scenarios were designed within the same seabed map, as shown in Figure 12. Scenario A uses the start point (10.0,10.0) and the goal point (290.0,190.0), while Scenario B uses the start point (10.0,190.0) and the goal point (290.0,10.0). The planned paths for the two scenarios are illustrated in Figure 13 and Figure 14, respectively. For each scenario, 30 independent trials were conducted under identical map settings, obstacle configuration rules, and algorithm parameters. The statistical results are summarized in Table 3, and the corresponding effect-size analysis is given in Table 4.

The statistical results are presented as mean ± standard deviation, median [interquartile range], and 95% confidence interval (CI) of the mean, respectively, based on 30 independent trials for each method in current-free scenarios without external disturbances. The mean ± standard deviation reflects the average performance and variability among repeated experiments, the median [interquartile range] indicates the typical performance and robustness against outliers, and the 95% CI represents the statistical reliability of the estimated mean value.

Since A* is a deterministic graph-search algorithm, repeated executions under identical terrain conditions, fixed start-goal configurations, and disturbance-free environments generate exactly the same optimal path, resulting in zero standard deviation and zero interquartile range. Therefore, the repeated runs of A* are retained as a deterministic reference baseline, whereas stochastic algorithms, including FWH, IFWH, IPSO, and SVF-RRT*, are evaluated according to their statistical variations over independent trials.

The statistical results demonstrate that IFWH achieves superior terrain adaptability and energy efficiency in current-free scenarios. In Scenario A, IFWH obtains the lowest average slope of 17.37°, reducing it by 43.91%, 47.24%, 44.94%, and 43.71% compared with A*, FWH, IPSO, and SVF-RRT*, respectively. Meanwhile, IFWH achieves the lowest propulsion energy cost of 1058.36 J, corresponding to reductions of 35.29%, 37.38%, 24.66%, and 35.93% compared with these methods.

In Scenario B, IFWH still obtains the lowest average slope of 13.11°, reducing it by 48.85%, 17.18%, 19.57%, and 39.97% compared with A*, FWH, IPSO, and SVF-RRT*, respectively. The estimated propulsion energy cost of IFWH is 1114.79 J, which is reduced by 26.01%, 41.31%, 0.43%, and 16.34% compared with these methods. Although IFWH produces a longer path than A*, IPSO, and SVF-RRT*, it achieves a substantially lower average slope and lower energy costs than most baseline methods. Its energy cost is comparable to that of IPSO in Scenario B.

The original FWH exhibits substantially longer paths and higher propulsion energy costs in Scenario B. This result indicates that FWH is more susceptible to local exploration oscillations when trajectory constraints become complex.

Overall, IFWH provides a better balance among terrain adaptability, path efficiency, and propulsion energy cost, demonstrating its effectiveness in current-free underwater terrain-aware path planning.

#### 5.1.3. Simulation Experiment of Superimposed Submarine Current

The second group of experiments superimposes spatially varying current fields on the static coral-reef terrain. Two representative current patterns are considered: a directional current and a rotary current. The directional current has a prescribed flow direction, whereas the rotary current is generated by randomly assigning the vortex center within the workspace.

**Figure 15:**
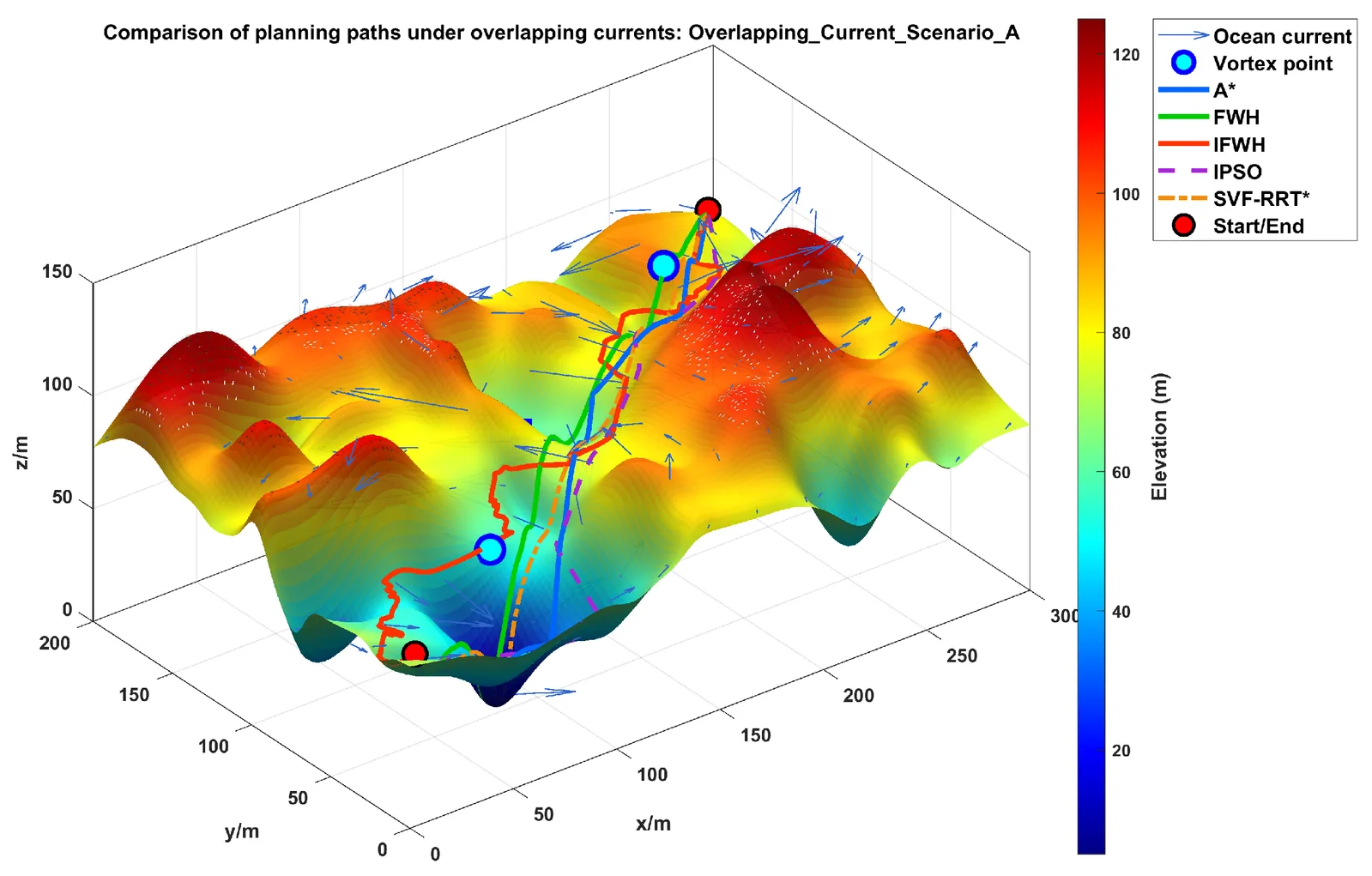
Overlapping-current Scenario A.

**Figure 16:**
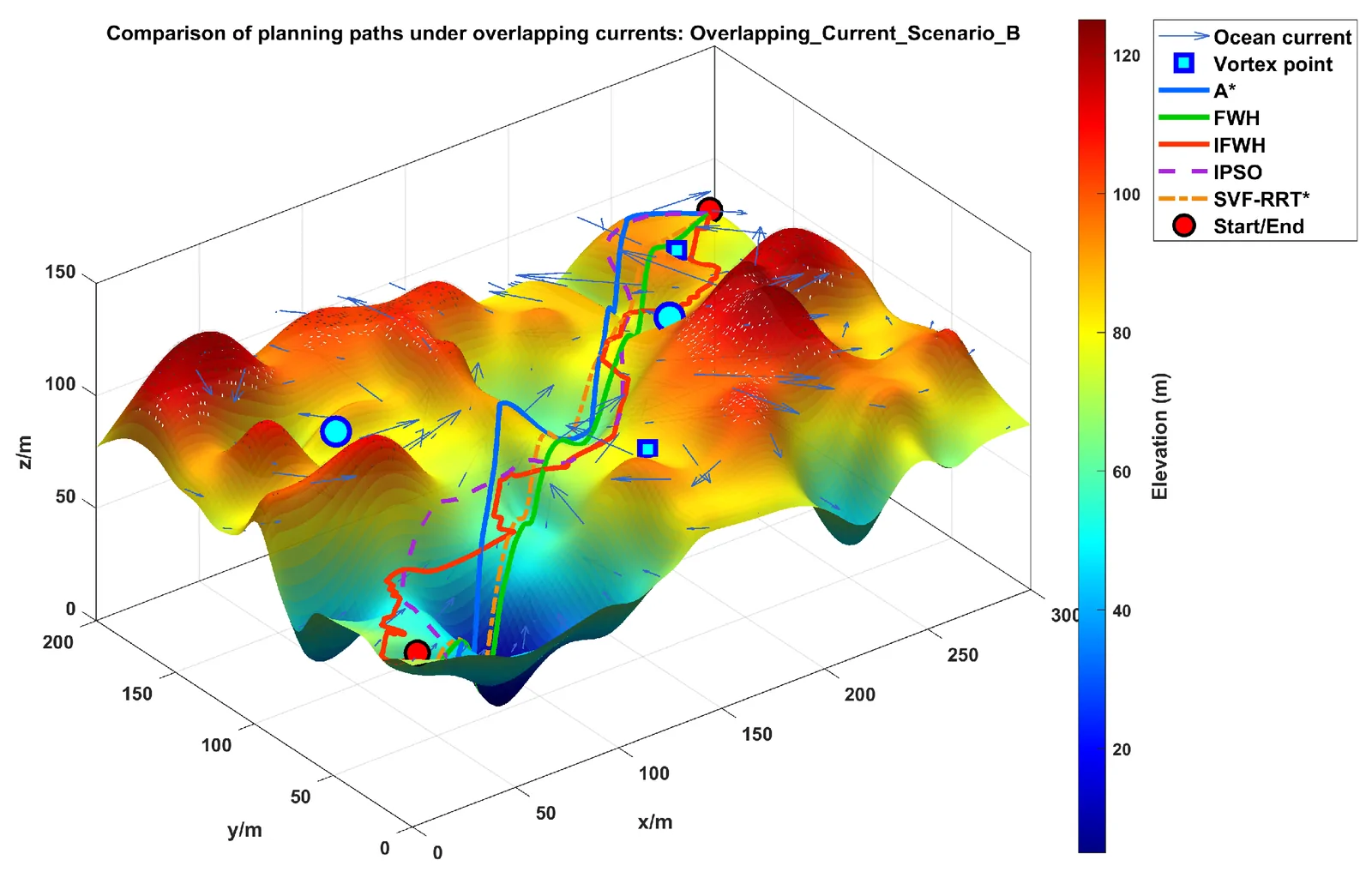
Overlapping-current Scenario B.

To improve the reliability of the evaluation under overlapping seabed-current conditions, each experimental setting was independently repeated 30 times. In each scenario, A*, FWH, IFWH, IPSO, and SVF-RRT* were evaluated under identical terrain configurations and overlapping current fields. The 3D path trajectories for the two scenarios are illustrated in Figure 15 and Figure 16, respectively. The statistical results are summarized in Table 5, including the three-dimensional path length, average terrain slope, and estimated propulsion energy consumption. The corresponding effect-size analysis is presented in Table 6. All methods achieved a 100% planning success rate in both scenarios.

In Scenario A, IFWH obtains the lowest average slope angle of 15.58°, which is reduced by 46.63%, 51.54%, 15.78%, and 50.16%, respectively, compared with A*, FWH, IPSO, and SVF-RRT*. Although IPSO generates a shorter trajectory due to its global optimization characteristic, its average slope angle remains higher, resulting in increased terrain-following requirements. Benefiting from the terrain-aware histogram guidance mechanism, IFWH effectively avoids steep seabed regions and achieves the lowest estimated propulsion energy cost, which is lower than A*, FWH, IPSO, and SVF-RRT* by 23.81%, 30.38%, 4.64%, and 24.19%, respectively.

In Scenario B, IFWH also maintains the best terrain adaptability, achieving an average slope angle of 18.73°, which is reduced by 32.31%, 40.88%, 9.25%, and 39.05% compared with A*, FWH, IPSO, and SVF-RRT*, respectively. Similar to Scenario A, IPSO obtains a relatively shorter path length, while IFWH provides a smoother trajectory with lower slope variation and achieves the lowest propulsion energy cost. Compared with A*, FWH, IPSO, and SVF-RRT*, IFWH reduces the estimated propulsion energy cost by 18.97%, 27.45%, 5.75%, and 24.85%, respectively.

Overall, the results demonstrate that IFWH does not simply optimize path distance but provides a better trade-off between terrain adaptability and energy efficiency under overlapping seabed-current conditions. The significantly reduced slope angles and lower propulsion energy cost indicate that the proposed method can effectively avoid steep seabed regions and reduce unnecessary vertical maneuvers, which is beneficial for energy-constrained AUV path planning.

#### 5.1.4. Comprehensive Environmental Simulation Experiment

The third group of experiments investigates the most challenging mixed underwater simulation scenarios by simultaneously considering static obstacles, moving obstacles, and ocean-current disturbances. Unlike the previous seabed-map experiments, which mainly focus on terrain adaptability and slope avoidance, this experiment evaluates the path feasibility, dynamic obstacle threat response, current disturbance effects, and local path-update behavior of the proposed method in offline simulations. To provide a unified geometric representation, both static and moving obstacles are modeled as spherical objects rather than being directly incorporated into the previous elevation-based terrain maps. This representation facilitates collision detection, minimum-clearance calculation, and statistical analysis of path-update events.

In these scenarios, the orange and purple spheres represent static and moving obstacles, respectively.The parameters of the moving obstacles are generated at the beginning of each trial using the reported random seed. Once initialized, each obstacle follows a deterministic sinusoidal trajectory throughout that trial. The position of moving obstacle *k* is defined as

$$p_{k}\left(t\right)=p_{k,0}+A_{k}\sin\left(\omega_{k}t+\phi_{k}\right)d_{k},$$

where $A_{k}$, $\omega_{k}$, $\phi_{k}$, and $d_{k}$ denote the amplitude, angular frequency, initial phase, and unit motion direction, respectively.

The simulation workspace is defined as a cubic region of 100×100×100 m. The ocean-current field is discretized using a 21×21×21 grid with a spacing of 5 m. The AUV starts from (5,5,5) m and aims to reach the goal position at (95,95,95) m. Each environment contains 15 static obstacles and 15 moving obstacles, with an obstacle radius of 5 m. For collision evaluation, the AUV is approximated as a sphere with a radius of 1 m. Furthermore, a desired clearance of 2 m is introduced as a soft constraint in the objective function, where insufficient clearance increases the path cost, while an actual collision occurs only when the surface clearance becomes non-positive.

To evaluate the influence of ocean currents on path planning performance, two representative current conditions are considered: a following-current condition and a rotary-current condition. The former represents a relatively regular flow field, whereas the latter describes a more complex spatially varying current environment. For each current condition, 30 independent trials are performed to quantitatively evaluate the performance of the proposed IFWH algorithm.

**Figure 17:**
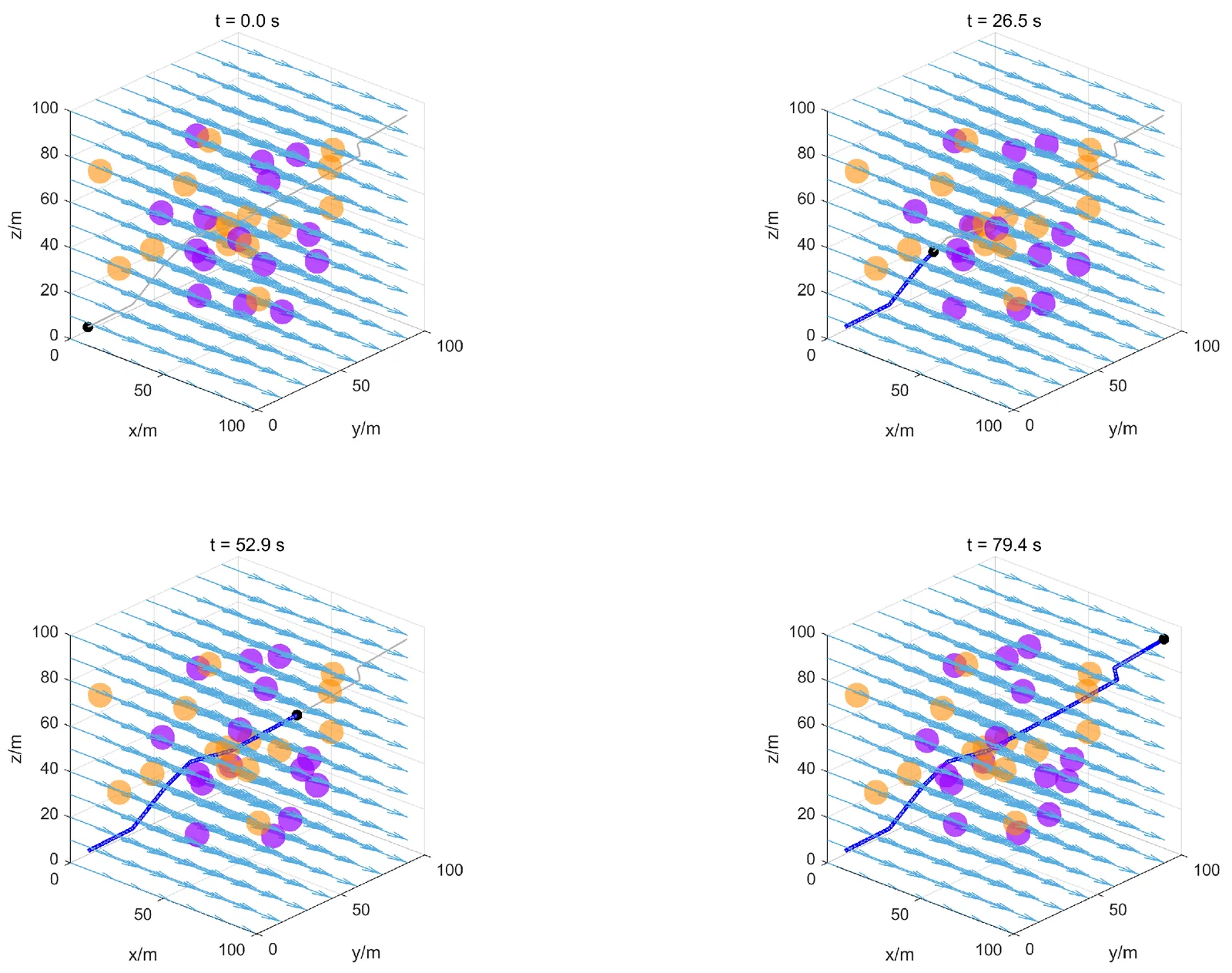
Time-lapsesnapshots under the following-current condition. Orange spheres represent static obstacles, purple spheres denote moving obstacles, blue arrows indicate the ocean-current direction, and the dark-blue solid line is the AUV planned path.

**Figure 18:**
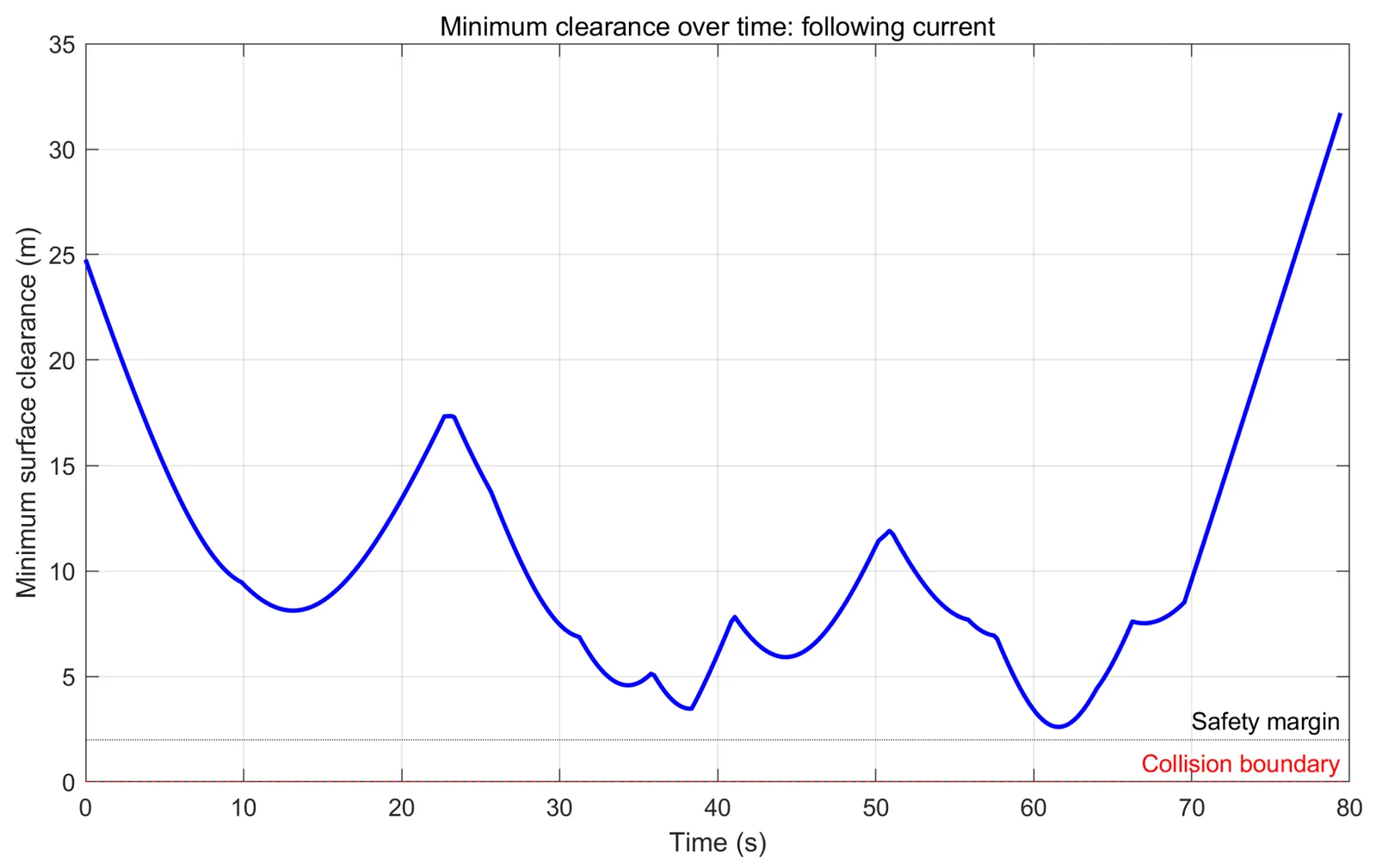
Minimum surface clearance over time under the following-current condition. Dark-blue solid line: instantaneous minimum clearance to obstacles; blue dashed line: predefined soft safety distance; red dashed line: dangerous distance threshold.

**Figure 19:**
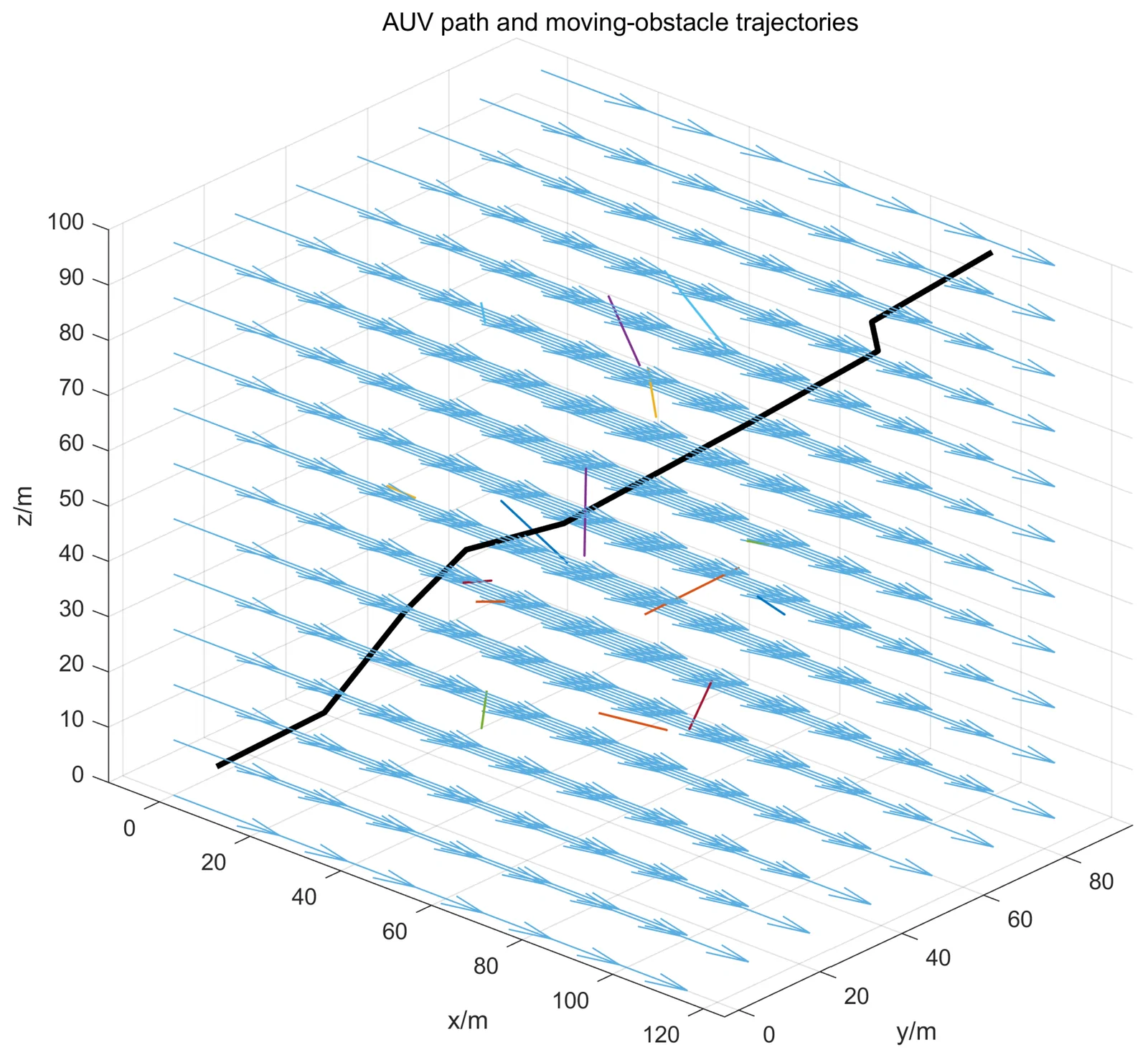
AUV and moving-obstacle trajectories under the following-current condition. Solid blue line: trajectory of the AUV; different coloured dashed lines represent the motion trajectories of individual moving obstacles.

**Figure 20:**
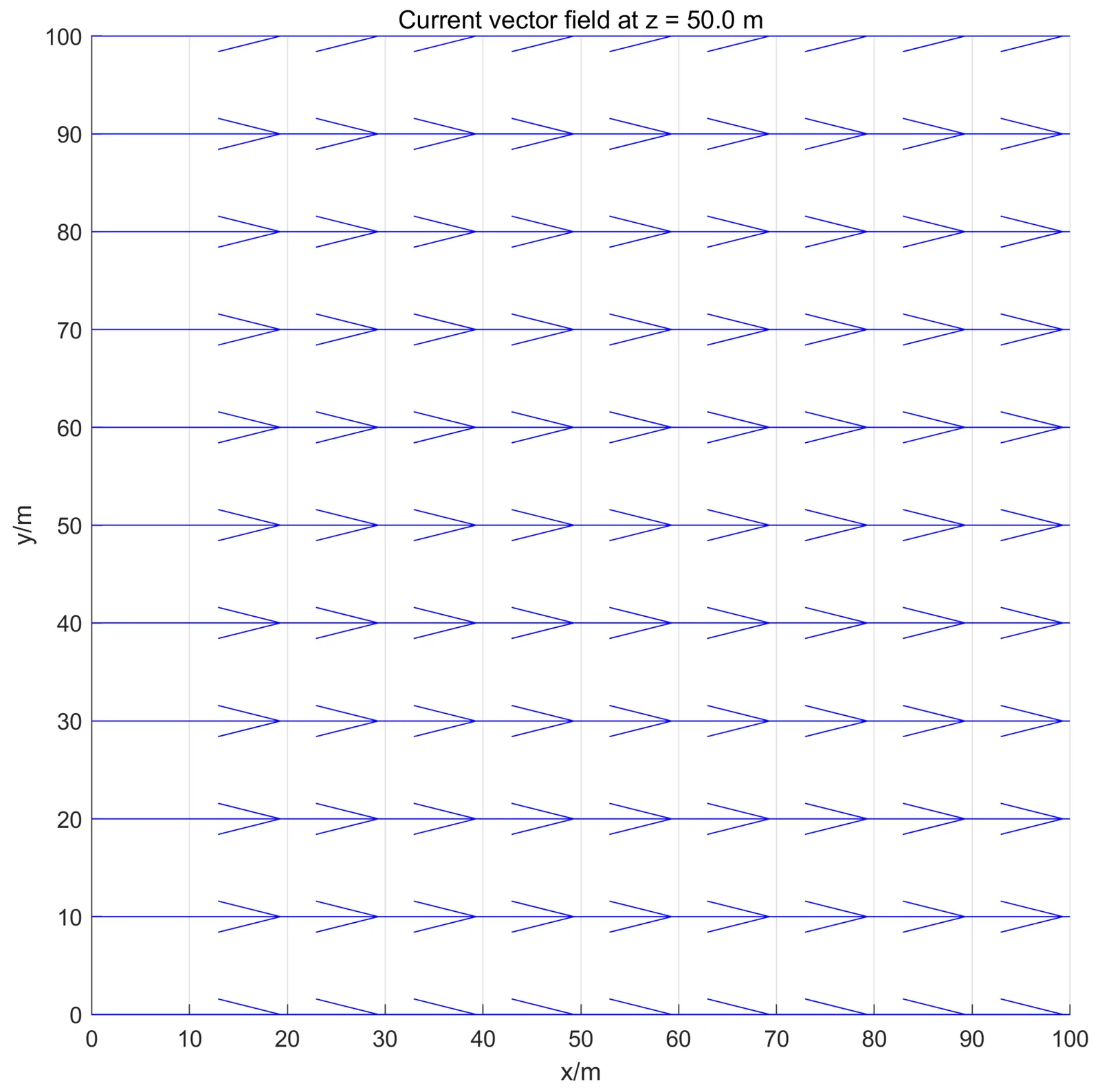
Current vectors at the middle-depth plane under the following-current condition.

**Figure 21:**
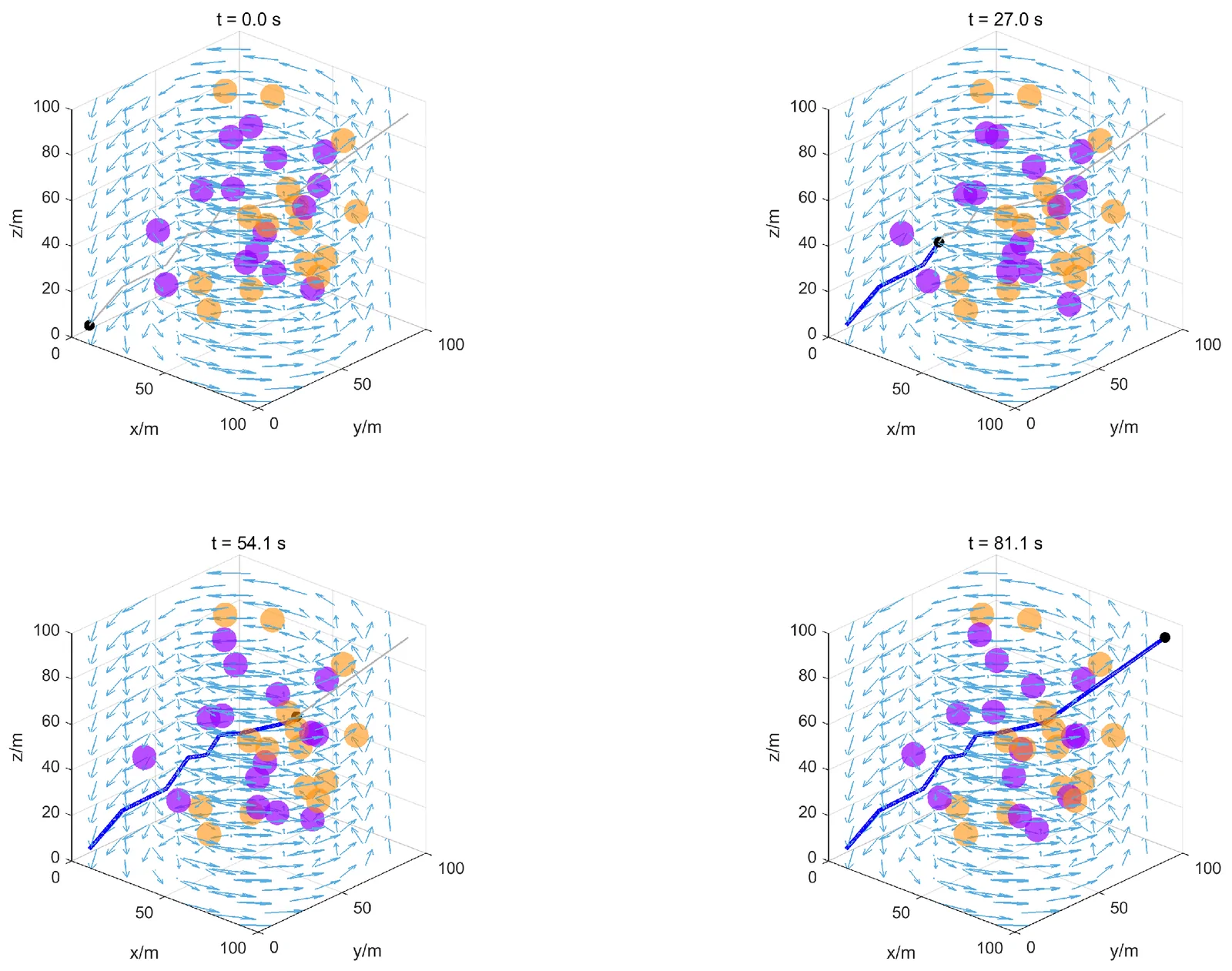
Time-Lapse snapshots under the rotary-current condition. Orange spheres represent static obstacles, purple spheres denote moving obstacles, blue arrows indicate the ocean-current direction, and the dark-blue solid line is the AUV planned path.

**Figure 22:**
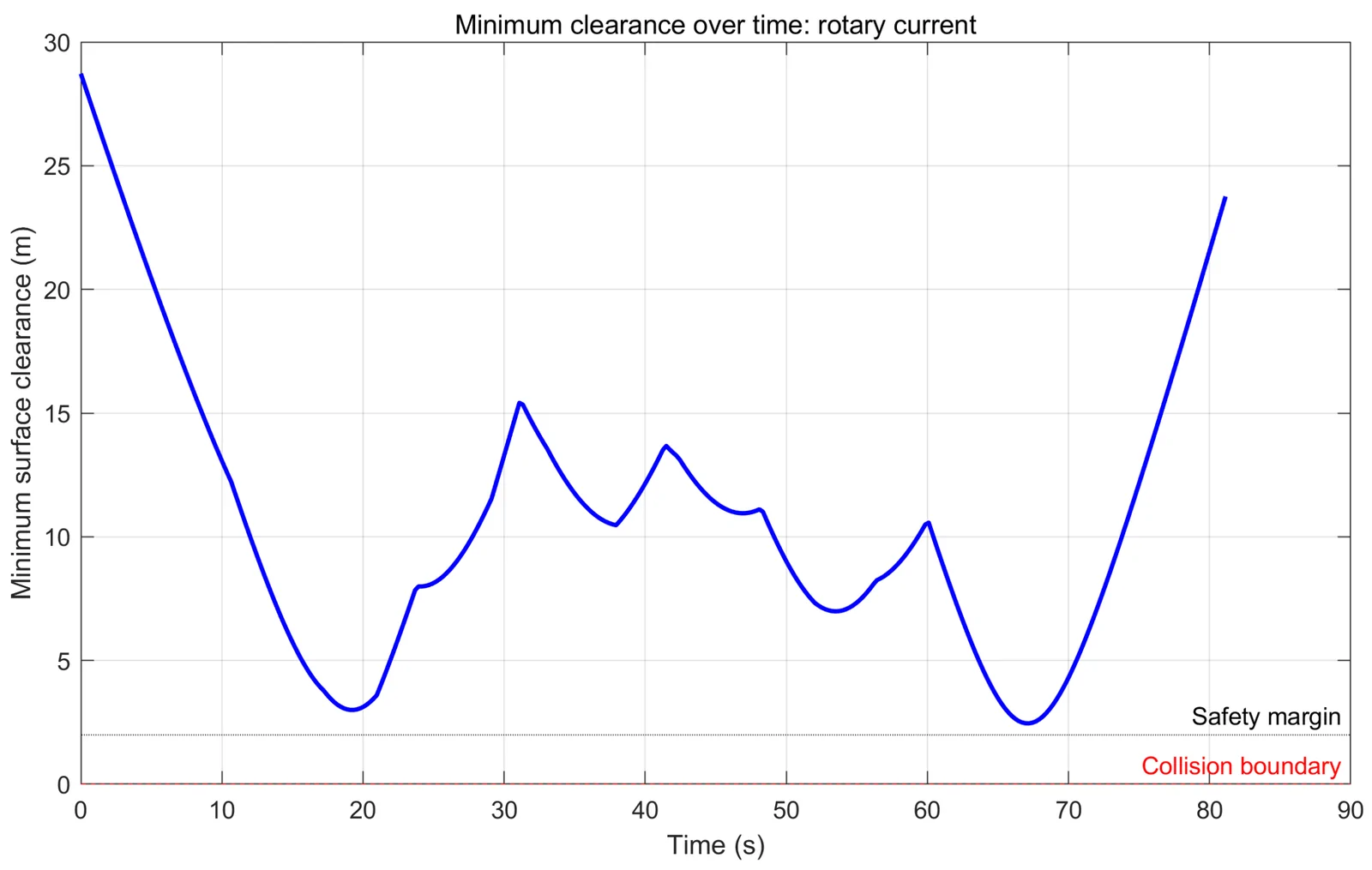
Minimum surface clearance over time under the rotary-current condition. Dark-blue solid line: instantaneous minimum clearance to obstacles; blue dashed line: predefined soft safety distance; red dashed line: dangerous distance threshold.

**Figure 23:**
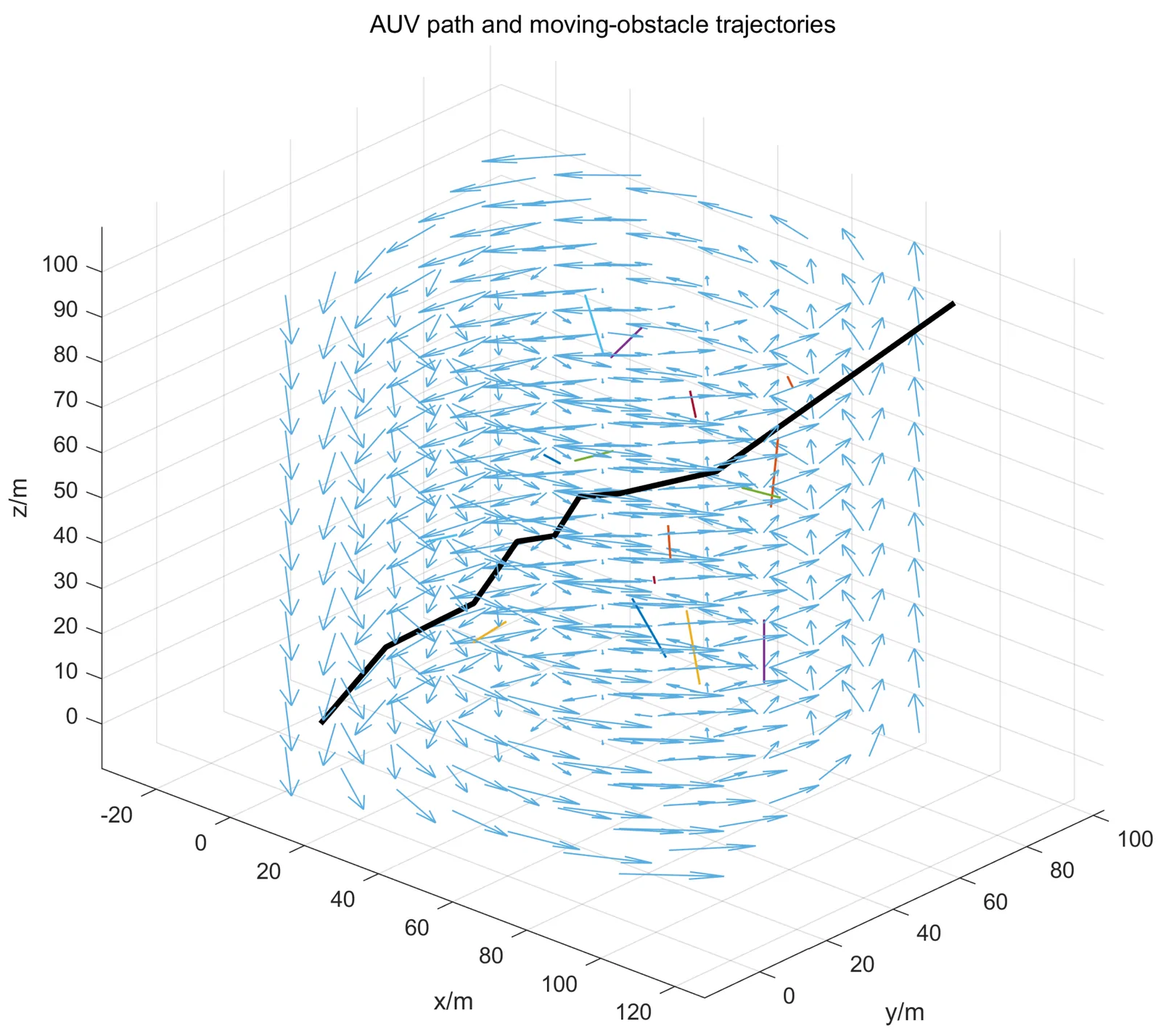
AUV and moving-obstacle trajectories under the rotary-current condition. Solid blue line: trajectory of the AUV; different coloured dashed lines represent the motion trajectories of individual moving obstacles.

**Figure 24:**
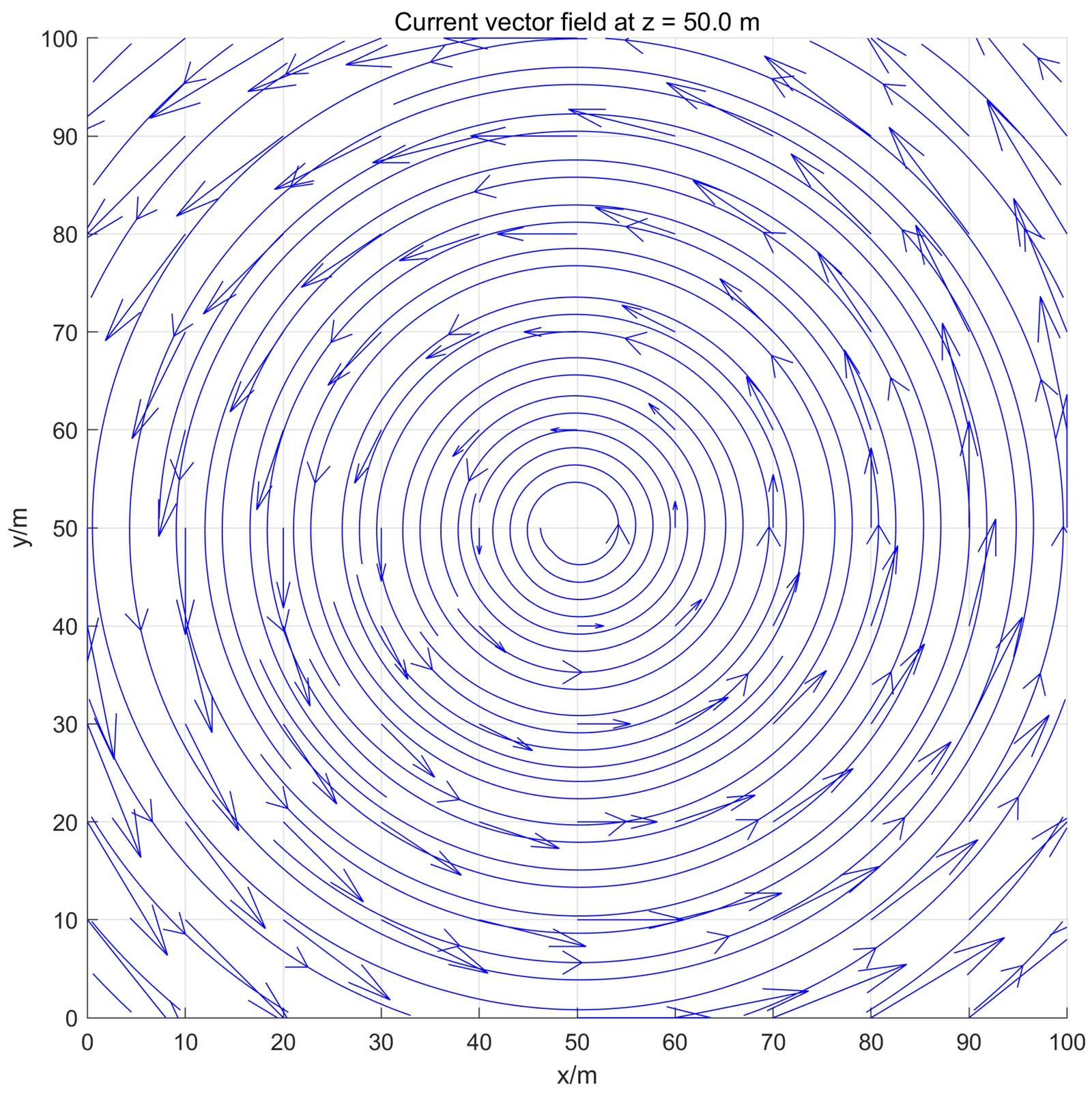
Current vectors and streamlines at the middle-depth plane under the rotary-current condition.

**Figure 25:**
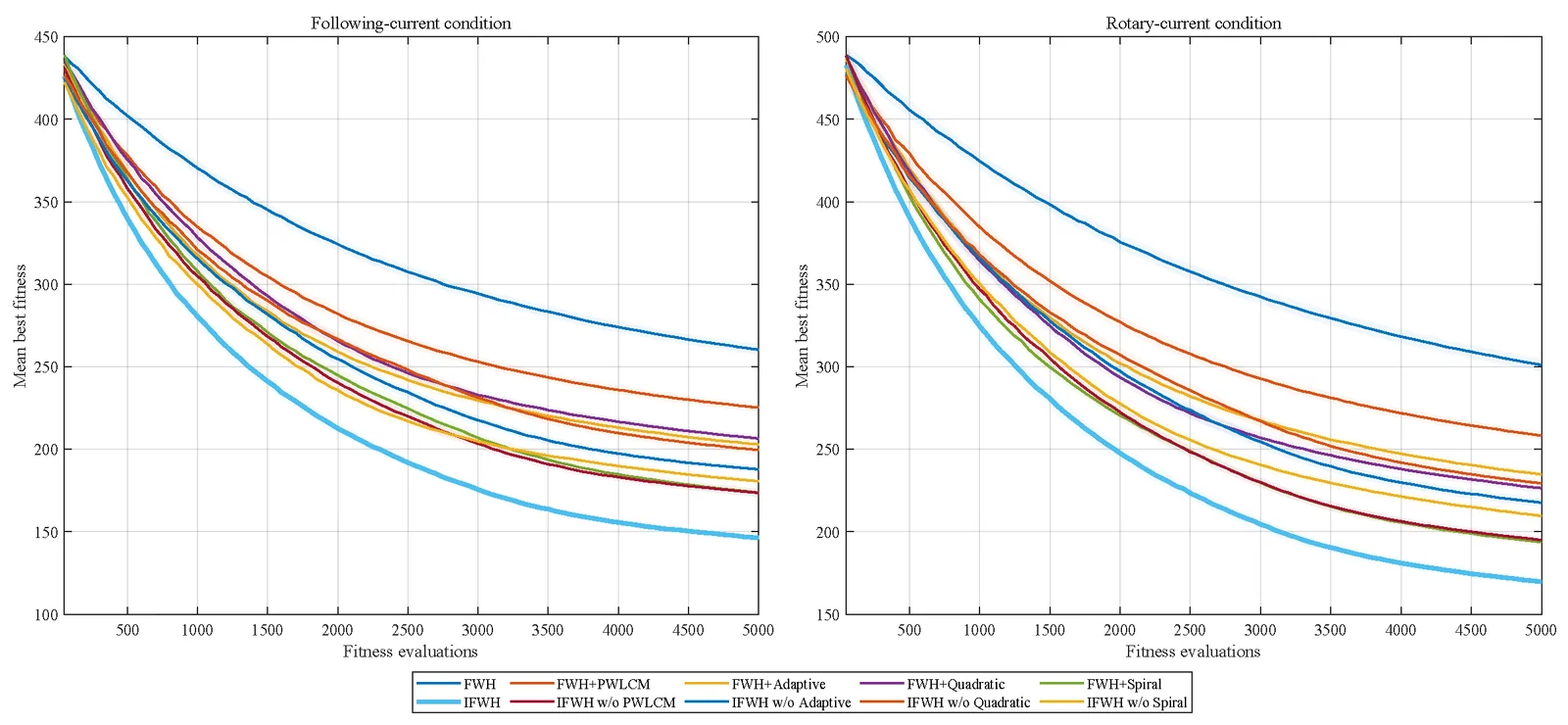
Convergence curves of the component-level ablation variants under following-current and rotary-current conditions.

Snapshots, clearance curves, trajectory illustrations, and flow-field visualizations for the following-current scenario are presented in Figure 17, Figure 18, Figure 19, and Figure 20, respectively. Corresponding results under the rotary-current condition are shown in Figure 21, Figure 22, Figure 23, and Figure 24. Convergence curves for ablation variants are given in Figure 25. The following-current field is spatially uniform and is defined as

$$v_{c}\left(x,y,z\right)=\left(0.60,0,0\right)\;m/s.$$

The rotary-current field rotates around the horizontal center of the workspace and is defined as

$$v_{c}\left(x,y,z\right)=\frac{0.60}{\sqrt{2}}\left(-\frac{y-50}{50},\frac{x-50}{50},0\right)\;m/s.$$

Its magnitude is zero at the center of the workspace and increases radially to a maximum of 0.60m/s at the horizontal corners. The vertical current component is zero.

The final path is resampled at spatial intervals no greater than 0.5m. The timestamp of each sampled AUV position is calculated according to the segment length, nominal through-water speed, and local current component along the direction of travel. At every resulting timestamp, the positions of all moving obstacles are updated using their prescribed trajectories.

The surface clearance between the AUV and obstacle *k* is calculated as

$$c_{k}\left(t\right)={∥p_{\mathrm{AUV}}\left(t\right)-p_{k}\left(t\right)∥}_{2}-\left(r_{\mathrm{AUV}}+r_{k}\right).$$

A run is classified as collision-free only when the AUV connects the prescribed start and goal positions and the minimum surface clearance remains positive at every sampled timestamp. The desired clearance of 2m is implemented as a soft optimization margin rather than a strict collision boundary.

Quantitative statistical results for the mixed-obstacle scenarios are summarized in Table 7.

The quantitative results are summarized in Table 7.

IFWH completed all runs without collision under both current conditions. The worst time-resolved surface clearances were 2.090 m and 2.068 m for the following- and rotary-current cases, respectively. The mean planning times were 1.674±0.700s and 0.692±0.053s. These success rates are empirical results under the reported obstacle-generation rules and random seeds rather than a guarantee for arbitrary environments.

Table 8 shows that the four mechanisms contribute to different aspects of IFWH. Compared with FWH, the complete IFWH reduces path length, average slope angle, and estimated energy consumption by 15.13%, 32.24%, and 24.95% under the following current, and by 14.54%, 31.49%, and 24.38% under the rotary current. Its success rate reaches 100% in both conditions.

Among the single-component variants, PWLCM mainly improves population robustness, adaptive transfer consistently improves the three path-quality metrics, and spiral refinement provides the largest individual reduction in path length and energy. Quadratic interpolation shows a stronger contribution under the rotary current. Removing this component increases path length, slope angle, and energy by 10.23%, 24.33%, and 16.2%, respectively, indicating that it is particularly useful in nonlinear local search regions. However, no single component fully reproduces the performance of IFWH, supporting the complementary roles of the four mechanisms. The complete IFWH increases computation time by less than 8% while using the same fitness-evaluation budget.

## 6. Conclusions

This study develops an improved fixed-width histogram algorithm named IFWH for simulation-driven AUV path planning in coral-reef underwater environments. Within a fixed-dimensional estimation-of-distribution framework, IFWH integrates PWLCM chaotic initialization, quadratic interpolation, adaptive global–local search switching, and logarithmic-spiral local refinement. A multi-constraint fitness function is constructed to evaluate path length, predicted propulsion energy, obstacle threat, terrain collision risk, as well as heading and slope variations. The proposed approach is positioned as a dynamics-informed geometric path planner, where the AUV dynamic model provides physical support for cost-function formulation, while full nonlinear vehicle dynamics and actuator states are not explicitly propagated during optimization.

For terrain experiments without ocean currents, IFWH achieves an effective balance among path length, terrain adaptability, and propulsion energy cost. In Scenario A, IFWH reduces the average slope by 43.91%, 47.24%, 44.94%, and 43.71% compared with A*, FWH, IPSO, and SVF-RRT*, respectively. Meanwhile, the estimated propulsion energy consumption is reduced by 35.29%, 37.38%, 24.66%, and 35.93%, respectively. In Scenario B, IFWH obtains the lowest average slope of 13.11° and reduces it by 48.85%, 17.18%, 19.57%, and 39.97% compared with A*, FWH, IPSO, and SVF-RRT*, respectively. The corresponding propulsion energy cost is 1114.79 J, which decreases by 26.01%, 41.31%, 0.43%, and 16.34%, respectively. Although some optimization-based methods generate shorter paths, IFWH achieves smoother terrain-following trajectories with improved energy efficiency.

Under overlapping seabed-current conditions, IFWH maintains robust performance and achieves feasible trajectories over 30 independent trials. The proposed method obtains average slope angles of 15.58° and 18.73° in the two current-disturbed scenarios, with estimated propulsion energy costs of 1163.57 J and 1116.99 J, respectively. These results indicate that IFWH can effectively adapt trajectory generation to complex terrain-current interactions while reducing unnecessary vertical maneuvers.

Experiments involving static obstacles, dynamic obstacles, and time-varying ocean currents further demonstrate the feasibility of IFWH under the considered simulation settings. All trials remain collision-free under both following-current and rotary-current conditions, with average planning times of 1.674±0.700 s and 0.692±0.053 s, respectively. However, these results are limited to the adopted simulation environments, obstacle generation strategies, parameter configurations, and random seeds, and do not represent guaranteed safety in arbitrary underwater scenarios.

The current study focuses on offline trajectory planning rather than a complete real-time autonomous navigation system. Nevertheless, the proposed IFWH framework has potential value as a high-level trajectory generation module within a hierarchical AUV autonomy architecture. Specifically, IFWH can provide globally optimized reference trajectories that simultaneously consider terrain adaptability, energy efficiency, obstacle avoidance, and maneuverability constraints, which may serve as informative guidance for subsequent low-level tracking and control layers. Such reference trajectories could potentially be integrated with nonlinear model predictive controllers (NMPC), adaptive controllers, or learning-based control policies to achieve closed-loop trajectory execution while considering vehicle dynamics, actuator constraints, and external disturbances.

Furthermore, the optimization characteristics of IFWH may provide useful prior knowledge for data-driven control approaches. For example, IFWH-generated trajectories could be used as expert demonstrations for imitation learning or as initialization strategies for reinforcement learning, thereby reducing inefficient exploration and improving training efficiency. In practical missions, the combination of IFWH-based global planning with real-time local replanning and state estimation modules may enable adaptive responses to dynamic environmental changes.

However, these potential integrations have not been experimentally validated in the present study. The current results are limited to offline MATLAB R2021a simulations and do not include closed-loop tracking, onboard computation, sensor uncertainty, hydrodynamic model mismatch, communication latency, or actuator saturation effects. Future research will therefore focus on hardware-in-the-loop validation, physical AUV experiments, integration with low-level trajectory tracking controllers, and the development of closed-loop autonomous planning and control frameworks.

## Figures and Tables

**Table 1:** Computational complexity of the evaluated algorithms.

Method	Computational Complexity
A^*^	O(VlogV).
FWH	$O\;\left(T[N_{p}D+N_{p}\log N_{p}+DH_{b}+N_{p}SM]\right)$.
IFWH	$O\left(N_{p}D\right)+O\;\left(T[N_{p}D+N_{p}\log N_{p}+DH_{b}+N_{p}SM+M_{d}]\right)$.
IPSO	$O\;\left(T_{p}N_{s}\left[D+SM\right]\right)$.
SVF-RRT^*^	$O(R^{2}+RSM)$.

*V*: grid nodes; $N_{p}$: FWH/IFWH population size; $N_{s}$: IPSO swarm size; *D*: trajectory dimension; *T*: FWH/IFWH iterations; $T_{p}$: IPSO iterations; $H_{b}$: histogram intervals; *S*: path segments; *M*: obstacles; $M_{d}$: dynamic obstacles; *R*: generated SVF-RRT^*^ nodes.

**Table 2:** Fixed parameter settings used in the comparative experiments.

Method	Parameter Settings
A^*^	Eight-connected grid; current coefficient: 0.30.
FWH	Step: 3.5 m; headings: 72; goal tolerance: 3.0 m; maximum iterations: 3500; stagnation limit: 60; current coefficient: 4.00.
IFWH	No current: $w_{d}=1.00$, $w_{s}=18.00$, $w_{z}=2.50$, $w_{e}=0.015$, $w_{r}=8.00$, $w_{h}=0.65$. Current: $w_{s}=35.00$, $w_{z}=4.50$, $w_{e}=0.020$, $w_{r}=12.00$, $w_{q}=0.80$, $w_{c}=5.00$, $w_{h}=0.55$.
IPSO	Waypoints: 8; swarm: 50; iterations: 140; ω:0.90→0.35; $c_{1}:2.50\to 0.50$; $c_{2}:0.50\to 2.50$; velocity ratio: 0.12.
SVF-RRT^*^	Nodes: 5000; step: 7.0 m; goal radius: 10.0 m; goal bias: 0.20; rewiring radius: 24.0 m; corridor bias: 0.35; corridor deviation: 5.0 m.
Common	Grid resolution: 2.0 m; AUV speed: 1.6 m/s; maximum current speed: 0.6 m/s.

**Table 3:** Statistical results of different methods in current-free scenarios.

Scenario	Method	Trials	Length3D (m)	Avg. slope (°)	Energy (J)
Scenario A	A*	30	438.33 ± 0.00 438.33 [0.00] 95% CI: [438.33, 438.33]	30.97 ± 0.00 30.97 [0.00] 95% CI: [30.97, 30.97]	1635.54 ± 0.00 1635.54 [0.00] 95% CI: [1635.54, 1635.54]
Scenario A	FWH	30	418.80 ± 20.51 412.99 [27.61] 95% CI: [411.60, 426.27]	32.92 ± 4.45 32.91 [4.84] 95% CI: [31.32, 34.46]	1690.22 ± 249.07 1724.99 [414.73] 95% CI: [1603.41, 1775.20]
Scenario A	IFWH	30	404.79 ± 25.53 407.83 [35.33] 95% CI: [395.46, 413.61]	17.37 ± 6.59 17.05 [9.09] 95% CI: [14.99, 19.70]	1058.36 ± 395.87 1132.46 [660.19] 95% CI: [918.59, 1200.41]
Scenario A	IPSO	30	374.56 ± 18.92 375.75 [18.98] 95% CI: [367.70, 381.07]	31.55 ± 5.81 31.63 [6.89] 95% CI: [29.53, 33.61]	1404.82 ± 214.77 1448.94 [342.48] 95% CI: [1328.11, 1480.91]
Scenario A	SVF-RRT*	30	433.84 ± 24.15 436.59 [37.24] 95% CI: [425.27, 442.21]	30.86 ± 5.23 31.11 [6.67] 95% CI: [28.99, 32.59]	1651.91 ± 245.56 1678.11 [205.89] 95% CI: [1568.70, 1737.45]
Scenario B	A*	30	449.25 ± 0.00 449.25 [0.00] 95% CI: [449.25, 449.25]	25.63 ± 0.00 25.63 [0.00] 95% CI: [25.63, 25.63]	1506.65 ± 0.00 1506.65 [0.00] 95% CI: [1506.65, 1506.65]
Scenario B	FWH	30	552.75 ± 48.45 558.88 [62.95] 95% CI: [535.21, 568.92]	15.83 ± 3.31 14.61 [5.00] 95% CI: [14.66, 17.00]	1899.38 ± 353.61 1926.75 [390.72] 95% CI: [1776.57, 2022.53]
Scenario B	IFWH	30	468.78 ± 23.29 469.15 [37.75] 95% CI: [460.70, 477.33]	13.11 ± 4.47 12.57 [6.93] 95% CI: [11.55, 14.73]	1114.79 ± 238.42 1132.94 [378.24] 95% CI: [1027.11, 1196.87]
Scenario B	IPSO	30	431.03 ± 22.34 433.00 [36.20] 95% CI: [423.03, 438.83]	16.30 ± 5.62 16.75 [7.51] 95% CI: [14.35, 18.26]	1119.59 ± 228.55 1112.19 [298.35] 95% CI: [1040.92, 1197.85]
Scenario B	SVF-RRT*	30	417.57 ± 15.05 420.04 [23.71] 95% CI: [412.16, 422.77]	21.84 ± 5.98 21.69 [8.81] 95% CI: [19.65, 23.95]	1332.55 ± 224.00 1362.65 [331.47] 95% CI: [1250.23, 1407.50]

**Table 4:** Statistical effect size analysis of IFWH compared with baseline methods in current-free scenarios.

Scenario	Comparison	Length3D	Avg. slope	Energy
Scenario A	IFWH vs. A*	δ=−0.933[−1.000,−0.800], $p_{\mathrm{Holm}}<0.001$	δ=−1.000[−1.000,−1.000], $p_{\mathrm{Holm}}<0.001$	δ=−0.867[−1.000,−0.667], $p_{\mathrm{Holm}}<0.001$
Scenario A	IFWH vs. FWH	δ=−0.293[−0.551,0.004], $p_{\mathrm{Holm}}=0.024$	δ=−0.962[−1.000,−0.898], $p_{\mathrm{Holm}}<0.001$	δ=−0.833[−0.944,−0.684], $p_{\mathrm{Holm}}<0.001$
Scenario A	IFWH vs. IPSO	δ=0.642[0.398,0.844], $p_{\mathrm{Holm}}<0.001$	δ=−0.909[−0.980,−0.804], $p_{\mathrm{Holm}}<0.001$	δ=−0.520[−0.747,−0.262], $p_{\mathrm{Holm}}<0.001$
Scenario A	IFWH vs. SVF-RRT*	δ=−0.604[−0.807,−0.367], $p_{\mathrm{Holm}}<0.001$	δ=−0.898[−0.973,−0.787], $p_{\mathrm{Holm}}<0.001$	δ=−0.827[−0.942,−0.673], $p_{\mathrm{Holm}}<0.001$
Scenario B	IFWH vs. A*	δ=0.600[0.267,0.867], $p_{\mathrm{Holm}}=0.006$	δ=−1.000[−1.000,−1.000], $p_{\mathrm{Holm}}<0.001$	δ=−0.933[−1.000,−0.800], $p_{\mathrm{Holm}}<0.001$
Scenario B	IFWH vs. FWH	δ=−0.869[−0.982,−0.700], $p_{\mathrm{Holm}}<0.001$	δ=−0.364[−0.636,−0.073], $p_{\mathrm{Holm}}=0.030$	δ=−0.944[−0.993,−0.864], $p_{\mathrm{Holm}}<0.001$
Scenario B	IFWH vs. IPSO	δ=0.744[0.551,0.889], $p_{\mathrm{Holm}}<0.001$	δ=−0.353[−0.624,−0.062], $p_{\mathrm{Holm}}=0.038$	δ=0.016[−0.271,0.307], $p_{\mathrm{Holm}}=0.932$
Scenario B	IFWH vs. SVF-RRT*	δ=0.942[0.844,1.000], $p_{\mathrm{Holm}}<0.001$	δ=−0.760[−0.907,−0.576], $p_{\mathrm{Holm}}<0.001$	δ=−0.498[−0.727,−0.238], $p_{\mathrm{Holm}}=0.004$

**Table 5:** Statistical Scenario B. Results under overlapping seabed-current conditions.

Scenario	Method	Trials	Length3D (m)	Avg. slope (°)	Energy (J)
Scenario A	A*	30	433.38 ± 10.81 433.40 [12.93] 95% CI: [429.41, 437.01]	29.19 ± 2.34 28.90 [2.36] 95% CI: [28.36, 30.01]	1527.11 ± 120.07 1541.68 [137.36] 95% CI: [1484.00, 1569.59]
Scenario A	FWH	30	427.03 ± 9.12 427.64 [12.79] 95% CI: [423.76, 430.21]	32.15 ± 2.25 32.03 [1.99] 95% CI: [31.36, 32.95]	1671.39 ± 113.93 1671.99 [151.04] 95% CI: [1631.14, 1711.72]
Scenario A	IFWH	30	442.12 ± 18.43 441.28 [24.14] 95% CI: [435.75, 448.71]	15.58 ± 2.61 15.71 [3.54] 95% CI: [14.67, 16.49]	1163.57 ± 84.49 1161.61 [121.67] 95% CI: [1133.20, 1192.95]
Scenario A	IPSO	30	398.96 ± 13.07 396.23 [15.85] 95% CI: [394.56, 403.65]	18.50 ± 2.72 18.84 [3.55] 95% CI: [17.57, 19.51]	1220.14 ± 121.49 1217.74 [132.31] 95% CI: [1178.52, 1263.24]
Scenario A	SVF-RRT*	30	421.06 ± 11.00 421.62 [12.66] 95% CI: [417.34, 425.03]	31.26 ± 2.90 30.71 [4.55] 95% CI: [30.25, 32.27]	1534.85 ± 173.88 1543.34 [252.55] 95% CI: [1474.26, 1593.49]
Scenario B	A*	30	424.19 ± 12.81 428.40 [16.60] 95% CI: [419.46, 428.59]	27.67 ± 3.07 28.04 [4.52] 95% CI: [26.59, 28.76]	1378.43 ± 90.04 1379.87 [140.43] 95% CI: [1347.17, 1410.22]
Scenario B	FWH	30	421.03 ± 9.09 418.54 [15.24] 95% CI: [417.76, 424.27]	31.68 ± 2.28 31.41 [1.73] 95% CI: [30.95, 32.55]	1539.56 ± 86.71 1538.01 [112.85] 95% CI: [1508.98, 1570.05]
Scenario B	IFWH	30	440.85 ± 21.43 437.34 [18.23] 95% CI: [433.58, 448.44]	18.73 ± 3.07 18.49 [4.47] 95% CI: [17.63, 19.79]	1116.99 ± 100.73 1099.78 [156.70] 95% CI: [1082.64, 1151.50]
Scenario B	IPSO	30	408.19 ± 14.60 408.61 [14.86] 95% CI: [403.00, 413.54]	20.64 ± 3.44 20.00 [4.43] 95% CI: [19.43, 21.81]	1185.10 ± 143.13 1215.89 [199.68] 95% CI: [1134.65, 1234.84]
Scenario B	SVF-RRT*	30	420.13 ± 11.50 419.54 [17.09] 95% CI: [416.24, 424.26]	30.73 ± 3.05 30.61 [3.43] 95% CI: [29.67, 31.79]	1486.34 ± 136.01 1475.90 [137.40] 95% CI: [1438.84, 1535.55]

**Table 6:** Statistical effect size analysis of IFWH compared with baseline methods in overlapping seabed-current scenarios.

Scenario	Comparison	Length3D	Avg. slope	Energy
Scenario A	IFWH vs. A*	δ=0.454[0.071,0.768], $p_{\mathrm{Holm}}=0.061$	δ=−1.000[−1.000,−1.000], $p_{\mathrm{Holm}}=0.001$	δ=−0.873[−0.962,−0.701], $p_{\mathrm{Holm}}<0.001$
Scenario A	IFWH vs. FWH	δ=0.690[0.363,0.905], $p_{\mathrm{Holm}}=0.003$	δ=−1.000[−1.000,−1.000], $p_{\mathrm{Holm}}=0.001$	δ=−0.842[−0.951,−0.672], $p_{\mathrm{Holm}}<0.001$
Scenario A	IFWH vs. IPSO	δ=0.956[0.841,1.000], $p_{\mathrm{Holm}}<0.001$	δ=−0.686[−0.923,−0.376], $p_{\mathrm{Holm}}=0.003$	δ=−0.385[−0.751,0.028], $p_{\mathrm{Holm}}=0.061$
Scenario A	IFWH vs. SVF-RRT*	δ=0.867[0.656,0.996], $p_{\mathrm{Holm}}<0.001$	δ=−1.000[−1.000,−1.000], $p_{\mathrm{Holm}}=0.001$	δ=−0.742[−0.901,−0.531], $p_{\mathrm{Holm}}<0.001$
Scenario B	IFWH vs. A*	δ=0.665[0.329,0.897], $p_{\mathrm{Holm}}=0.004$	δ=−1.000[−1.000,−1.000], $p_{\mathrm{Holm}}=0.001$	δ=−0.821[−0.943,−0.612], $p_{\mathrm{Holm}}<0.001$
Scenario B	IFWH vs. FWH	δ=0.755[0.458,0.953], $p_{\mathrm{Holm}}=0.001$	δ=−1.000[−1.000,−1.000], $p_{\mathrm{Holm}}=0.001$	δ=−0.914[−0.978,−0.762], $p_{\mathrm{Holm}}<0.001$
Scenario B	IFWH vs. IPSO	δ=0.974[0.893,1.000], $p_{\mathrm{Holm}}<0.001$	δ=−0.381[−0.708,−0.002], $p_{\mathrm{Holm}}=0.001$	δ=−0.355[−0.733,0.071], $p_{\mathrm{Holm}}=0.141$
Scenario B	IFWH vs. SVF-RRT*	δ=0.776[0.510,0.957], $p_{\mathrm{Holm}}=0.001$	δ=−1.000[−1.000,−1.000], $p_{\mathrm{Holm}}=0.001$	δ=−0.781[−0.921,−0.554], $p_{\mathrm{Holm}}<0.001$

**Table 7:** Quantitative results of IFWH in mixed static- and moving-obstacle environments.

Metric	Following Current	Rotary Current
Successful runs	30/30	30/30
Path length (m)	225.331±62.610	161.255±4.652
Energy (J)	1090.191±375.476	920.213±27.030
Mean minimum clearance (m)	2.658	2.949
Worst clearance (m)	2.090	2.068
Planning time (s)	1.674±0.700	0.692±0.053

**Table 8:** Component-Level ablation results of IFWH under different current conditions.

Current	Variant	Success (%)	Length (m)	Slope (°)	Energy (J)	Time (s)	Evaluations
Following	FWH	93.3	204.82±18.46	32.41±2.85	978.34±112.65	2.431±0.182	5000
Following	FWH+PWLCM	96.7	198.36±16.72	30.86±2.51	932.47±96.28	2.466±0.176	5000
Following	FWH+Adaptive	96.7	190.45±14.63	27.92±2.13	864.53±82.16	2.498±0.184	5000
Following	FWH+Quadratic	96.7	190.06±14.92	29.32±2.26	875.43±86.17	2.507±0.188	5000
Following	FWH+Spiral	100.0	182.67±12.34	24.83±1.76	798.46±68.54	2.526±0.169	5000
Following	IFWH	100.0	173.84±8.92	21.96±1.28	734.28±49.63	2.618±0.173	5000
Following	IFWH w/o PWLCM	96.7	181.73±13.16	24.72±1.89	781.35±71.46	2.574±0.181	5000
Following	IFWH w/o Adaptive	96.7	186.42±14.38	26.15±2.04	816.72±78.35	2.552±0.177	5000
Following	IFWH w/o Quadratic	93.3	189.12±15.46	26.47±2.17	828.56±84.32	2.563±0.179	5000
Following	IFWH w/o Spiral	96.7	184.37±13.82	25.47±1.96	802.64±74.53	2.541±0.186	5000
Rotary	FWH	86.7	216.38±22.41	34.26±3.18	1186.53±143.72	2.486±0.195	5000
Rotary	FWH+PWLCM	90.0	208.74±19.63	32.17±2.84	1124.36±126.48	2.519±0.187	5000
Rotary	FWH+Adaptive	93.3	201.16±17.28	29.13±2.37	1038.42±104.65	2.553±0.193	5000
Rotary	FWH+Quadratic	93.3	198.65±17.36	29.08±2.35	1018.42±108.63	2.574±0.196	5000
Rotary	FWH+Spiral	96.7	195.37±14.76	27.16±2.05	981.54±88.63	2.581±0.178	5000
Rotary	IFWH	100.0	184.92±10.14	23.47±1.52	897.31±61.48	2.684±0.185	5000
Rotary	IFWH w/o PWLCM	93.3	194.68±15.93	26.82±2.18	958.46±91.37	2.631±0.194	5000
Rotary	IFWH w/o Adaptive	93.3	199.53±17.24	28.36±2.31	1009.62±101.54	2.608±0.189	5000
Rotary	IFWH w/o Quadratic	93.3	203.84±18.72	29.18±2.46	1042.68±112.37	2.637±0.183	5000
Rotary	IFWH w/o Spiral	93.3	197.26±16.71	27.54±2.23	987.43±96.28	2.596±0.198	5000

## Data Availability

The original contributions presented in this study are included in the article. Further inquiries can be directed to the corresponding author.
